# Effect of Large Language Model–Powered Virtual Standardized Patients on History-Taking Among Undergraduate Medical Students: Propensity-Matched Cohort Study

**DOI:** 10.2196/92486

**Published:** 2026-09-09

**Authors:** Yuchen He, Chen Chen, Rong Yin, Wuyang Zhang, Haoli Chang, Wei Yang, Fei Li, Xinhua Li, Zhuying Xia, Xiaoyun Xie, Jing Huang, Qiuming Zeng, Guang Yang, Jing Wu

**Affiliations:** 1 Clinical Skills Training Center Xiangya Hospital Central South University Changsha, Hunan China; 2 Department of Nephrology Xiangya Hospital Central South University Changsha, Hunan China; 3 National Clinical Research Center of Geriatric Disorders Xiangya Hospital Central South University Changsha, Hunan China; 4 Teaching and Research Section of Diagnostics Xiangya Hospital Central South University Changsha, Hunan China; 5 Department of Endocrinology Xiangya Hospital Central South University Changsha, Hunan China; 6 Department of Respiratory Medicine Xiangya Hospital Central South University Changsha, Hunan China; 7 Department of Geriatric Medicine Xiangya Hospital Central South University Changsha, Hunan China; 8 Department of Rheumatology and Immunology Xiangya Hospital Central South University Changsha, Hunan China; 9 Department of Neurology Xiangya Hospital Central South University Changsha, Hunan China; 10 Clinical Research Center for Neuroimmune and Neuromuscular disorders Xiangya Hospital Central South University Changsha, Hunan China; 11 Department of General Medicine Xiangya Hospital Central South University Changsha, Hunan China; 12 Hunan Engineering Research Center for Obesity and its Metabolic Complications Xiangya Hospital Central South University Changsha, Hunan China

**Keywords:** artificial intelligence, education, large language model, medical history taking, medical, practice behaviors, real-time feedback, undergraduate, virtual standardized patients

## Abstract

**Background:**

Medical history taking (MHT) is a foundational clinical competency for medical students; however, traditional training models using standardized patients face challenges such as resource constraints. Large language model–powered virtual standardized patients (LLM-VSPs) offer a safe, repeatable platform for self-directed practice with AI-automated feedback. Nevertheless, their effectiveness in authentic teaching environments and underlying learning mechanisms require further investigation.

**Objective:**

This study aims to evaluate the impact of an LLM-VSP system as an extracurricular self-practice tool on undergraduate medical students’ MHT performance in an authentic educational setting without disrupting routine instruction, and to further explore potential associations among practice behaviors, baseline proficiency, and intervention effects.

**Methods:**

This prospective cohort study enrolled 168 third-year medical students. Based on voluntary participation, students were assigned to an intervention group (n=120, using LLM-VSP) or a control group (n=48, receiving routine instruction). Propensity score matching (PSM) balanced confounding factors, yielding 40 matched pairs. Baseline MHT performance was assessed via virtual patient examination after didactic instruction but before clinical practicum. The primary outcome was end-of-term MHT performance assessed at an Objective Structured Clinical Examination station with real standardized patients. The differences between groups were compared using independent samples *t* test, with robustness validated through multiple linear regression, sensitivity analyses, and Rosenbaum bounds analyses. Exploratory analyses investigated the association between practice behaviors and scores, and observed benefit differences across baseline levels.

**Results:**

After PSM, baseline characteristics were balanced (standardized mean difference <0.1). The intervention group exhibited higher total MHT scores than controls (mean 87.71, SD 7.29 vs mean 83.74, SD 8.06; mean difference 3.98, 95% CI 0.55-7.40 points; *P*=.02; with a medium effect size of Cohen *d*=0.52). Advantages were observed in content (*P*=.03) and communication skills (*P*=.04) subscores. Regression analysis confirmed robust intervention effects (*B*=3.924, 95% CI 1.90-5.94; *P*<.001; *R*^2^=0.708), with sensitivity analyses supporting reliability. Exploratory analysis suggested that mere practice behavior metrics were not independent predictors of final scores, potentially being constrained by baseline proficiency, implying a “cognitive threshold” for effective AI-assisted training. Subgroup analysis indicated a trend of differential benefits: high baseline students appeared to show larger gains (matched sample: mean difference 5.93, 95% CI 2.17-9.70; overall sample: mean difference 7.04, 95% CI 3.84-10.25), whereas improvements in medium and low baseline students were relatively limited (matched sample: mean difference 2.94-2.99; overall sample: mean difference 1.29-1.64).

**Conclusions:**

Introducing LLM-VSPs as a self-practice tool in diagnostics education may help improve undergraduate medical students’ MHT performance. Preliminary evidence suggests a potential “cognitive threshold,” implying students with solid theoretical foundations and higher baseline proficiency may better achieve skill transformation through AI-assisted autonomous practice. This provides a basis for future stratified teaching strategies and differentiated guidance for students of varying baseline levels.

## Introduction

Medical history taking (MHT) is an essential practical skill for clinicians in the diagnostic and treatment process [1], and is also an important learning objective of the *diagnostics* course. MHT, as a core practical clinical competency, requires repeated deliberate practice for its mastery [2]. Traditional MHT teaching predominantly relies on 2 primary pedagogical approaches: bedside teaching involving real patients and standardized patient (SP)–based teaching [3-5]. Yet the aforementioned instructional process remains plagued by an array of challenges and inherent limitations. Specifically, bedside teaching with real patients struggles to accommodate large-scale educational demands and carries the potential risk of exacerbating doctor-patient tensions; in contrast, SP-based teaching is confronted with issues such as substantial training costs, cumbersome management processes, difficulties in ensuring instructional standardization and homogeneity, and inadequacies in simulating authentic, complex clinical symptoms and signs [6-8].

Virtual standardized patients (VSPs) refer to virtual teaching tools constructed using digital technologies that can simulate the clinical characteristics of real patients and provide interactive feedback [9], used for the training and assessment of medical students’ clinical diagnosis and treatment skills and communication abilities. VSPs can effectively address the limitations of SPs while exhibiting characteristics of repeatability and cost-effectiveness, providing new instructional modalities for medical education [10,11]. However, early VSPs exhibited rigid interaction and were difficult to simulate the natural language communication of real patients [12,13]. Large language model–powered virtual standardized patients (LLM-VSPs) can deliver more naturalistic and authentic interactive experiences. They can provide medical students with highly realistic clinical consultation scenarios and timely, individualized feedback [14,15], which endows them with great application potential in medical history collection, doctor-patient communication, and clinical reasoning training [16-19]. However, the quality of LLM prompts directly affects the output performance of VSPs [20]. Algorithmic biases and hallucinations in AI may lead to factually incorrect outputs [21], which poses challenges to the reliability of automated AI scoring. Moreover, unsupervised autonomous practice with LLM-VSPs may foster communication behaviors that violate clinical ethics. Prior research on companion chatbots has documented not only AI-initiated inappropriate behaviors but also the potential for users to direct offensive or dismissive language toward AI [22]. In educational contexts, such bidirectional misbehavior may erode rather than cultivate empathy, and risks reinforcing unprofessional communication habits.

Current research on the effectiveness of LLM-VSPs in diagnostic courses remains relatively scarce, particularly studies that conduct in-depth analysis of LLM-VSPs’ usage behaviors and the corresponding learning outcomes among medical students, as well as exploration of differential benefits among students with different abilities. Accordingly, large-scale blind integration of this tool into teaching is premature. To clarify the real effectiveness and optimal application methods of LLM-VSPs, we conducted a small-scale pilot empirical exploration without interfering with the regular undergraduate teaching process. This study aims to conduct a prospective cohort study using voluntary recruitment combined with propensity score matching (PSM) to investigate 3 core research questions: first, whether incorporating LLM-VSPs into the auxiliary practice of MHT can improve medical students’ proficiency in this skill; second, what the correlation is between students’ practice behaviors during LLM-VSPs interactions and their subsequent learning outcomes; third, what the differential benefits are among student subgroups with different abilities from this intervention. The findings of this study are intended to provide empirical evidence and optimization directions for the future application of AI in MHT skill training.

## Methods

### Creation of LLM-VSPs

**Figure 1:**
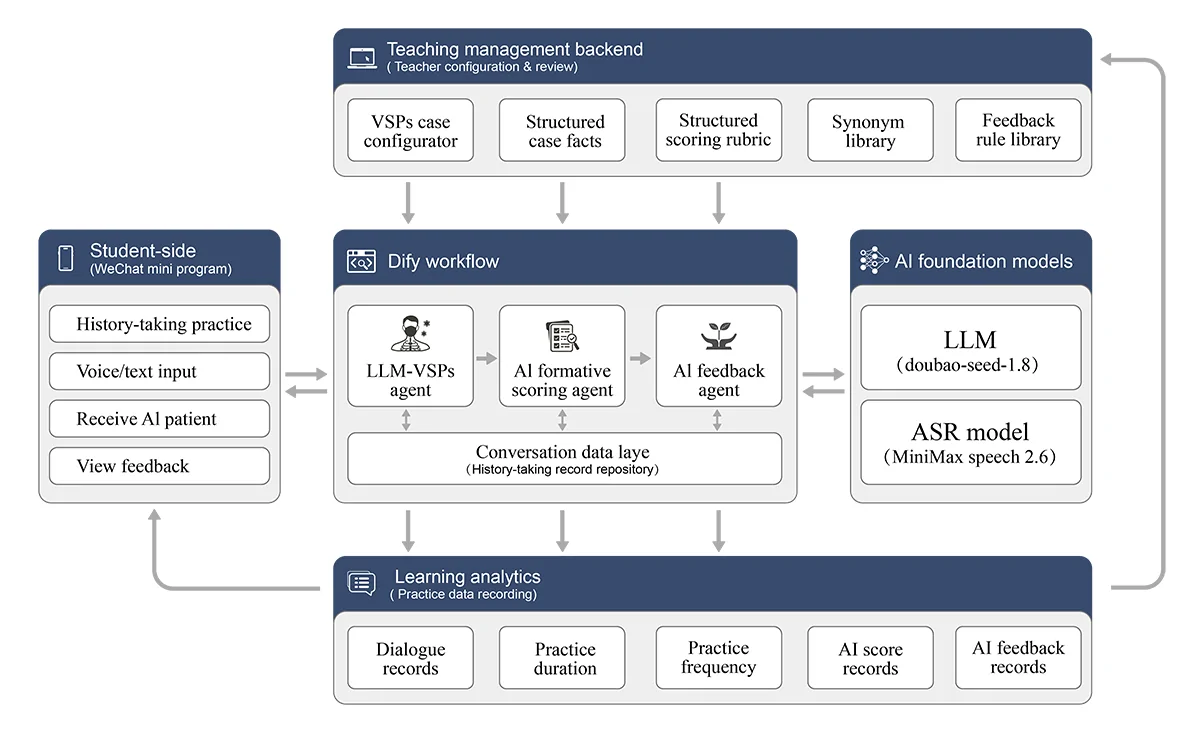
Architecture and workflow of the DoctorU large language model–powered virtual standardized patients (LLM-VSPs) system. ASR: automatic speech recognition; LLM: large language model; VSP: virtual standardized patient.

This study used the DoctorU teaching system for MHT practice. This system constructed a multiagent workflow including LLM-VSPs agent, AI formative scoring agent, and AI feedback agent (Figure 1). The LLM-VSPs agent (see Multimedia Appendix 1 for detailed prompts) was responsible for simulating patient roles. The AI formative scoring agent scored students’ single MHT performance based on a structured scoring rubric. After the MHT session, the system input the dialogue records, structured case facts (Multimedia Appendix 1), and corresponding structured scoring rubrics into the agent, which assessed whether students covered key MHT content through semantic judgment based on preset scoring points, score values, and scoring conditions, and generated scores, missed items, and brief evaluations. For synonymous questioning methods or nonstandard expressions, the system allowed semantic matching through a synonym library to determine whether students substantially addressed corresponding scoring points. The AI feedback agent generated immediate formative feedback after practice, combining dialogue records and scoring results, covering completed content, missed important information, deficiencies in MHT logic, and suggestions for subsequent improvement based on the feedback rule library. The system-maintained context within a single MHT session to ensure coherence of patient responses; however, individualized dialogue memories were not shared between different students and different training sessions to avoid cross-student information contamination. Simultaneously, the backend database logs the complete practice data of all students to support longitudinal tracking of individual learning growth and cross-sectional analysis of interaction performance and learning processes across students and cases. All system parameters remained fixed during the study period, and specific model invocation parameter settings are shown in Table S1 in Multimedia Appendix 1.

In accordance with the teaching syllabus and curriculum schedule of the diagnostic course for students in the 5-year clinical medicine program, and with reference to the textbook *Diagnostics (10th Edition)* published by the People’s Health Press, clinical cases were selected following a symptom-oriented approach. The criteria for case development were determined to primarily focus on newly admitted patients presenting with a single symptom, thereby ensuring uniformity in case difficulty levels. All cases did not involve real clinical patient data. Concurrently, a structured scoring rubric was developed, with a total score of 100 points. This rubric comprised 2 core dimensions: 80 points allocated to the completeness and accuracy of MHT content, and 20 points dedicated to the proficiency of MHT communication skills. Serving as a core functional module of the LLM-VSPs system, this rubric was designed to facilitate students’ completeness and logicality of MHT content through a cycle of “practice-assessment-feedback-correction.” Furthermore, given the existing constraints of AI systems in simulating nonverbal communication, fine-grained assessment of MHT interpersonal skills remained unfeasible [23]; therefore, the scoring weight leaned more heavily toward content items. A total of 13 cases were developed: 11 for student practice and 2 for baseline assessment (see Multimedia Appendix 2 for detailed cases and the scoring rubrics). Overall, 8 diagnostic course instructors of the current semester were invited to conduct a comprehensive review and refinement of all LLM-VSP cases, to safeguard the accuracy and instructional applicability of the cases.

### Participants

The participants of this study consisted of 276 third-year medical students enrolled in the *diagnostics* course at Xiangya Hospital of Central South University during the spring semester of the 2024-2025 academic year. These students were from 3 cohorts: the 2022 entry class of the 5-year clinical medicine program, the 2022 entry class of the 5-year stomatology program, and the 2022 entry class of the 5-year anesthesiology program. All participating students had no previous training experience in MHT skills prior to the study. The inclusion criteria for this study were defined as follows: (1) being enrolled in the *diagnostics* course during the current semester and (2) voluntarily participating in this study and providing informed consent. The exclusion criteria were established as follows: (1) failure to complete the baseline assessment of MHT skills at the start of the semester and (2) failure to complete the postintervention assessment of MHT skills at the end of the semester. Upon completion of the research, 108 students were excluded (107 students failed to complete the baseline test and 1 student failed to complete the final test), and finally 168 students were included in data analysis.

To determine student participation in the LLM-VSP practice sessions, a registration link was distributed to all students enrolled in this study. According to the principle of voluntary participation, all eligible students were assigned to one of the two groups: the experimental group (group A) received MHT training via LLM-VSPs–based practice, while the control group (group B) undertook conventional teaching for the same clinical skill training. However, according to the minimum effective practice criteria established in this study, among the students who had voluntarily registered for the LLM-VSPs–based practice group, 9 individuals failed to meet the aforementioned minimum practice threshold based on their practice records. They were deemed to have not effectively participated in the LLM-VSPs–based practice. According to the per-protocol analysis principle, the aforementioned students were reclassified into the control group. The final actual group allocation resulted in 120 students in the experimental group and 48 students in the control group (see Multimedia Appendix 3 for the detailed participant screening flowchart).

### Intervention Measures

In this study, students in group A used LLM-VSPs to practice history taking. In alignment with the curriculum schedule and teaching progress of the *diagnostics* course, starting from the third week of the semester, each student in group A was assigned 1 LLM-VSP–based clinical case per week, resulting in a total of 11 cases distributed throughout the intervention period. They could complete the autonomous practice of LLM-VSPs for history taking during their spare time through a mobile app. There were no restrictions on the number of practices and the duration of each practice. After each practice, the system would immediately generate AI-based assessment feedback. Students in group B did not use LLM-VSPs for the practice. Both groups of students received conventional teaching on MHT skills, including sessions with SPs and bedside practical training, in accordance with the predefined curriculum schedule of the *diagnostics* course.

To ensure the authenticity and effectiveness of the LLM-VSPs intervention, we invited diagnostic course instructors of the current semester to conduct simulation tests. The results indicated that skillfully completing a single MHT session comprising basic self-introduction, chief complaint, history of present illness, past medical history, personal history, and family history required a minimum duration of 4 minutes. Furthermore, according to the LLM-VSPs structured scoring rubric, inquiring once for each module of the MHT content, including self-introduction, patient general information, chief complaint, history of present illness, and past medical history, would achieve a total score of 25 points. The research team unanimously agreed that practice sessions failing to meet the aforementioned criteria could not achieve the training objectives of structured MHT logic and content completeness and were considered ineffective practice. Therefore, this study established the minimum effective practice criteria as a single practice session duration of ≥4 minutes and an AI score of ≥25 points.

### Data Collection

#### Baseline Characteristics

Demographic data, including sex and academic major, were retrieved from the university’s student administration records. Sex was recorded as male or female. After completing the theoretical course of MHT and the practical training course of *Medical Interview and Communication Skills* at the start of the semester, 2 case-based MHT tests were administered to all participants via the DoctorU instructional system. The baseline score for each student was determined using the average of their AI-generated assessment scores from these 2 tests.

#### Outcome Measures

At the end of the semester, a postintervention assessment of MHT skills was administered to all participating students. The assessment was carried out in the format of Objective Structured Clinical Examination (OSCE) stations, with real SPs serving as simulated patients for the history-taking interaction. The assessment comprised 4 stations, each corresponding to a distinct clinical case scenario: coronary heart disease, pneumonia, urinary tract infection, and hyperthyroidism. Notably, none of these scenarios had been included in the LLM-VSP practice cases library prior to the assessment. All participants were randomly assigned by computer to one of the 4 assessment stations to complete the MHT assessment. Two standardized and trained examiners independently scored each candidate’s performance. The final assessment score was the average of the scores assigned by the 2 examiners. The intraclass correlation coefficient (ICC) based on a 2-way random effects model (absolute agreement) was used to evaluate interrater reliability, which was calculated independently within each assessment case. The scoring rubric was provided by the Department of Diagnostic Medicine, with a total score of 100 (including 60 points for MHT content and 40 points for MHT skills). This rubric served as a summative assessment tool, aiming to comprehensively evaluate students’ comprehensive MHT abilities after systematic diagnostic course learning; therefore, the scoring weights for MHT content and skills were set relatively balanced (see Multimedia Appendix 4 for the specific scoring rubrics).

#### LLM-VSPs Practice Data of Group A

All students’ MHT practice data were recorded in detail via the backend of the DoctorU system. Unqualified practice data were excluded in accordance with the predefined minimum threshold for effective practice established earlier in the study. Ultimately, the LLM-VSPs–based MHT practice data of all students in group A were compiled, encompassing the following key metrics: the total number of effective practice sessions, the total number of effective practice cases, the average duration per practice session, the total duration of practice, the highest AI-generated assessment score, and the average AI-generated assessment score.

Statistics

Statistical analyses were performed using R software (version 4.4.2; R Foundation for Statistical Computing). PSM was implemented to mitigate selection bias arising from voluntary group allocation. Matching variables included sex, academic major, baseline test scores, and final test cases, with 1:1 nearest neighbor matching conducted between groups A and B using a caliper width of 0.1 SDs of the logit of the propensity score. Postmatching balance was evaluated through standardized mean differences (SMDs), where SMD<0.1 indicated adequate balance. Final MHT assessment score comparisons between matched groups used independent samples *t* test or *t’* test, and Cohen *d* effect size was calculated. Multivariate linear regression adjusted for covariates was used to confirm intervention effect robustness. To assess the potential impact of unmeasured confounding factors on the results, the Rosenbaum bounds (Γ) were calculated. To verify the robustness of the primary findings against protocol deviations, a sensitivity analysis was conducted following the intention-to-treat (ITT) principle. Specifically, the 9 students who failed to meet the practice threshold were retained in the intervention group according to their original allocation. PSM and subsequent comparative analyses (including independent samples *t* tests and multivariate linear regression) were repeated on this ITT sample to ensure the consistency of the conclusions.

An exploratory analysis was conducted within group A, using Pearson correlation analysis and partial correlation analysis (adjusted for baseline scores) to investigate the associations between practice behavior metrics and MHT scores, and a multivariate linear regression model was further constructed to explore the independent predictive effect of practice behaviors. The model included baseline scores as a covariate, and practice behavior metrics were selected based on the results of the preliminary correlation analyses.

To investigate the heterogeneity of intervention effects across baseline proficiency levels, a generalized linear model was constructed. This model incorporated an interaction term between group assignment and baseline scores (as a continuous variable), while adjusting for sex, academic major, and final test case. This analysis was conducted in both the matched and overall samples. Furthermore, students were divided into high-, medium-, and low-level subgroups based on the 33rd and 67th percentiles of baseline scores. An exploratory subgroup analysis was performed to describe the outcome distributions across these subgroups. The subgroup models were adjusted for final test cases (with the overall sample additionally adjusting for baseline scores), excluding sex and academic major. Results are presented as adjusted means with SEs.

All tests were 2-tailed with the significance threshold set at α=.05.

### Ethical Considerations

The study was reviewed by the Clinical Medical Ethics Review Committee of Xiangya Hospital of Central South University. The ethics committee reviewed the study in its entirety and concluded that the research was eligible for exemption from formal ethical review, as it complied with Specific Applicable Situations 1 and 2 of Article 32 of the Measures for Ethical Review of Life Science and Medical Research Involving Humans (2023 edition) issued by the National Health Commission of the People’s Republic of China [24]. An official ethical review comment letter was issued (ethics committee record number 2026081836).

Although the study was exempt from formal ethical review, comprehensive information regarding the research was fully provided, and written informed consent was secured from all participating students before enrollment.

All data were deidentified prior to analysis. No individual student data or identifiable information are disclosed in this manuscript.

Participants received no compensation.

## Results

### Baseline Characteristics of the Participants

**Figure 2:**
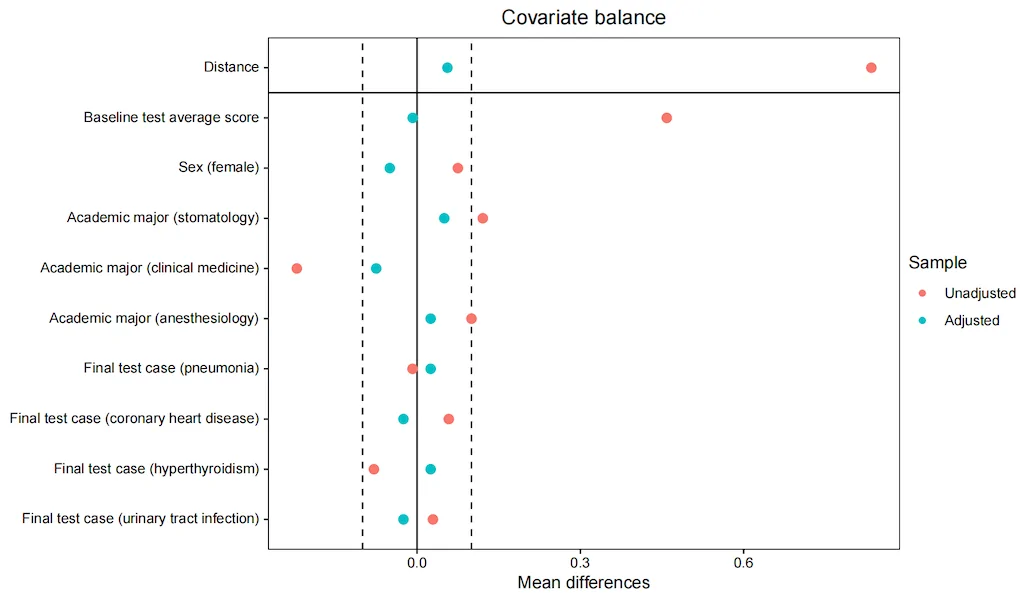
Propensity score matching Love plot.

Table 1 shows significant prematching differences between group A (n=120) and group B (n=48) in academic major distribution (*P*=.02) and baseline assessment scores (*P*=.01). Owing to computerized random station allocation, the distribution of final test cases was well-balanced between the two groups (*P*=.66). Following matching, 80 participants (40 in each group) were retained for final analysis. All SMDs between matched groups fell below 0.1, demonstrating balanced demographic characteristics (sex), academic majors, baseline test scores, and final test cases between the 2 groups (Figure 2).

**Table 1:** Baseline characteristics of participants before and after propensity score matching.

Variables		Before matching								After matching											SMD^a^
		Total (N=168)	Group A (n=120)	Group B (n=48)	Test statistic		*P* value		Total (n=80)		Group A (n=40)		Group B (n=40)		Test statistic		*P* value				
**Sex** **, n** **(** **%)**						0.54 (1)^b^		.46								0.06 (1)^b^		.81			
	Male	61 (36.3)	41 (34.2)	20 (41.7)					26 (32.5)		14 (35.0)		12 (30.0)						0.050		
	Female	107 (63.7)	79 (65.8)	28 (58.3)					54 (67.5)		26 (65.0)		28 (70.0)						–0.050		
**Academic** **major** **,** **n** **(** **%)**						7.74 (2)^b^		.02								—^c^		1.00			
	Stomatology	39 (23.2)	32 (26.7)	7 (14.6)					16 (20.0)		9 (22.5)		7 (17.5)						0.050		
	Clinical medicine	110 (65.5)	71 (59.2)	39 (81.3)					59 (73.8)		28 (70.0)		31 (77.5)						–0.075		
	Anesthesiology	19 (11.3)	17 (14.2)	2 (4.2)					5 (6.3)		3 (7.5)		2 (5.0)						0.025		
Baseline test score, mean (SD)		63.2 (11.2)	64.6 (10.8)	59.7 (11.5)	2.57 (166)^d^		.01		61.4 (10.3)		61.3 (9.2)		61.4 (11.4)		–0.04 (78)^d^		.97		0.008		
**Final** **test case** **, n** **(** **%)**						1.59 (3)^b^		.66								0.20 (3)^b^		.98			
	Pneumonia	48 (28.6)	34 (28.3)	14 (29.2)					23 (28.8)		12 (30.0)		11 (27.5)						0.025		
	CHD^e^	42 (25.0)	32 (26.7)	10 (20.8)					19 (23.8)		9 (22.5)		10 (25.0)						–0.025		
	Hyperthyroidism	36 (21.4)	23 (19.2)	13 (27.1)					19 (23.8)		10 (25.0)		9 (22.5)						0.025		
	UTI^f^	42 (25.0)	31 (25.8)	11 (22.9)					19 (23.8)		9 (22.5)		10 (25.0)						–0.025		

^a^SMD: standardized mean difference. SMD value of less than 0.1 is widely recognized in clinical research, educational evaluation, and social science studies as a critical threshold for excellent baseline balance between 2 comparison groups.

^b^Chi-square test (*df*).

^c^For “academic major” after matching, the *P* value was calculated using the Fisher exact test, which does not produce a test statistic (indicated by “—”).

^d^*t* test (*df*).

^e^CHD: coronary heart disease.

^f^UTI: urinary tract infection.

### Comparison of the Final MHT Assessment Scores

Interrater reliability analysis of the final MHT assessment scores revealed ICCs across all 4 station cases ranging from 0.65 to 0.80 (Table S1 in Multimedia Appendix 5), indicating moderate to good agreement between examiners across the different stations. The postmatching comparison results of the final test scores for MHT between the 2 groups after intervention are presented in Table 2. The average total score of group A in MHT was 3.98 points higher than that of group B (mean 87.71, SD 7.29 vs mean 83.74, SD 8.06; *P*=.02; mean difference 3.98, 95% CI 0.55-7.40; Cohen *d*=0.52). Group A also demonstrated statistically significant advantages over group B in both the MHT content subscore (*P*=.03) and the MHT skill subscore of the assessment (*P*=.04).

**Table 2:** Comparison of the final medical history-taking (MHT) test scores between the 2 groups after propensity score matching.

Variables	Total (n=80), mean (SD)	Group A (n=40), mean (SD)	Group B (n=40), mean (SD)	Mean Difference (95% CI)	*t* test (*df*)	*P* value	Cohen *d* (95% CI)
Total score of MHT (100 points)	85.72 (7.90)	87.71 (7.29)	83.74 (8.06)	3.98 (0.55-7.40)	2.31 (78)	.02	0.52 (0.06-0.97)
Score of MHT content (60 points)	48.80 (5.93)	50.23 (5.65)	47.38 (5.93)	2.85 (0.27-5.43)	2.20 (78)	.03	0.49 (0.04-0.94)
Score of MHT skills (40 points)	36.92 (2.49)	37.49 (2.13)	36.36 (2.70)	1.13 (0.04-2.21)	2.07 (74.0)	.04	0.46 (0.01-0.91)

### Robustness Test

Multivariate linear regression confirmed the robustness of the above results (Table 3). In the unadjusted model, group A demonstrated significantly higher total MHT scores than group B (*B*=3.975; *P*=.02). Following adjustment for covariates (baseline test scores, sex, academic major, and final test cases), the intervention effect remained statistically significant (*B*=3.924; *P*<.001). The model’s coefficient of determination *R*^2^ reached 0.708, indicating that the model explained the majority of the variance in final MHT assessment scores. In addition, to assess the potential threat of unmeasured confounding factors to the matching results, this study calculated the Rosenbaum bounds Γ=1.21 for this primary analysis sample.

**Table 3:** Multivariate linear regression analysis of the final medical history-taking test scores (n=80).

Model and variables			*B*^a^ (95% CI)	SE	*β* ^b^	*t* test (*df*)	*P* value	VIF^c^ value	
**Model 1 (without adjusting for covariates)^d^**									
	Constant		83.738 (81.318 to 86.157)	1.215	N/A^e^	68.898 (78)	<.001	N/A	
	Group A (reference group=group B)		3.975 (0.553 to 7.397)	1.719	0.253	2.313 (78)	.02	N/A	
**Model 2 (adjusted for covariates)^f^**									
	Constant		84.919 (77.770-92.068)	3.585	N/A	23.686 (71)	<.001	N/A	
	Group A (reference group=group B)		3.924 (1.904 to 5.944)	1.013	0.250	3.873 (71)	<.001	1.007	
	Baseline test scores (points)		0.012 (–0.089 to 0.114)	0.051	0.016	0.245 (71)	.81	1.039	
	Female (reference group=male)		3.481 (1.200 to 5.761)	1.144	0.208	3.043 (71)	.003	1.065	
	**Academic** **major** **(** **reference** **g** **roup=** **stomatology** **)**								1.040
		Clinical medicine	2.911 (0.233 to 5.589)	1.343	0.163	2.168 (71)	.03		
		Anesthesiology	–0.528 (–5.196 to 4.140)	2.341	–0.016	–0.226 (71)	.82		
	**Final** **test case (reference group** **=** **pneumonia** **)**								1.041
		CHD^g^	–15.854 (–18.765 to –12.942)	1.460	–0.860	–10.856 (71)	<.001		
		Hyperthyroidism	–4.289 (–7.174 to –1.405)	1.447	–0.233	–2.965 (71)	.004		
		UTI^h^	–6.745 (–9.552 to –3.938)	1.408	–0.366	–4.792 (71)	<.001		

^a^Unstandardized coefficient.

^b^Standardized coefficient.

^c^VIF: variance inflation factor.

^d^Model parameters: *R*^2^=0.064; Radj^2^=0.052; *F*_1, 78_=5.348; *P*=.02.

^e^N/A: not applicable.

^f^Model parameters: *R*^2^=0.708; Radj^2^=0.675; *F*_8,71_=21.552; *P*<.001.

^g^CHD: coronary heart disease.

^h^UTI: urinary tract infection.

### Sensitivity Analysis

According to the ITT analysis principle, the 9 students who were reassigned to the control group for failing to meet the practice threshold were reincluded in the intervention group (group A with 129 students and group B with 39 students), and PSM was reperformed to obtain 33 matched pairs. The analysis results are presented in Table S2 in Multimedia Appendix 5, revealing that group A’s MHT total scores remained significantly higher than those of group B (mean difference 5.27, 95% CI 1.52-9.02; *P*=.01; Cohen *d*=0.70). The multivariate linear regression results after adjusting for covariates remained significant (*B*=5.190, 95% CI 2.505-7.875; *P*<.001). In addition, the Rosenbaum bounds Γ value for this sensitivity sample increased from 1.21 in the primary analysis to 1.42.

### Exploratory Analysis of LLM-VSPs Practice Data and the Final MHT Test Scores.

Table S3 in Multimedia Appendix 5 summarizes the valid LLM-VSPs practice data for students in group A. On average, participants completed 6.25 valid practice sessions covering 5.21 unique clinical cases. Preliminary Pearson correlation analyses revealed that without adjustment for baseline scores, only total duration of practice (*r*=0.183; *P*=.046) and the average AI-generated assessment score (*r*=0.233; *P*=.01) exhibited weak positive linear associations with final MHT test scores. All other practice metrics failed to demonstrate statistically significant linear correlations with the outcome. To exclude baseline scores as an important confounding factor, we further conducted partial correlation analysis (Table S3 in Multimedia Appendix 5). The results showed that after controlling for baseline scores, the correlations of both the total duration of practice (*r_partial_*=0.075; *P*=.42) and the average AI-generated assessment score (*r_partial_*=0.097; *P*=.29) with final MHT test scores disappeared. Further multiple linear regression analysis confirmed (Table 4) that after adjusting for baseline, neither the total duration of practice (*β*=.058; *P*=.56) nor the average AI-generated assessment score (*β*=.096; *P*=.38) could independently predict final test scores, whereas baseline scores showed a marginal predictive trend (*β*=.209; *P*=.06). Although the overall regression model showed statistical significance (*F*_3, 116_=3.947; *P*=.01), the adjusted coefficient of determination Radj^2^=0.069 was at a low level.

**Table 4:** Multiple linear regression analysis predicting the final medical history-taking (MHT) test scores in group A (n=120).^a^

Variables	*B* (95% CI)	SE	*β*	*t* test (*df*)	*P* value
Constant	71.011 (60.515 to 81.508)	5.300	N/A^b^	13.400 (116)	<.001
Baseline test scores (points)	0.146 (–0.004 to 0.296)	0.076	0.209	1.922 (116)	.06
Total duration of LLM-VSPs^c^–based MHT practice sessions (minutes)^d^	0.006 (–0.015 to 0.027)	0.010	0.058	0.582 (116)	.56
Average AI-generated assessment score for LLM-VSPs–based MHT^d^	0.076 (–0.093 to 0.246)	0.086	0.096	0.891 (116)	.38

^a^Model parameters: *R*^2^=0.093; Radj^2^=0.069; *F*_3, 116_=3.947; *P*=.01.

^b^N/A: not applicable.

^c^LLM-VSP: large language model–powered virtual standardized patient.

^d^The practice behavior metrics were selected as predictors based on their significant associations observed in the preliminary unadjusted correlation analyses.

### Baseline Heterogeneity Tests and Subgroup Analyses

Results from the interaction term analysis regarding baseline heterogeneity suggested that the interaction between group assignment and baseline status did not reach statistical significance in either the matched or overall samples (matched sample: *P*=.63; overall sample: *P*=.20; see Tables S4 and S5 in Multimedia Appendix 5). However, the main effect of group assignment indicated that students in the LLM-VSP group had significantly higher final exam scores than those in the control group in both the matched sample (mean difference 3.92, SE 1.02, 95% CI 1.89-5.95; *P*<.001) and the overall sample (mean difference 3.61, SE 0.99, 95% CI 1.65-5.57; *P*<.001). Exploratory subgroup analyses (Table 5) indicated that the between-group mean difference in the high baseline subgroup appeared to be 5.93 (95% CI 2.17-9.70) in the matched sample and 7.04 (95% CI 3.84-10.25) in the overall sample. In contrast, the mean differences observed in the low and moderate baseline subgroups appeared relatively smaller (matched sample: 2.94-2.99; overall sample: 1.29-1.64).

**Table 5:** Exploratory descriptive analysis of the final medical history-taking test scores by baseline tertiles in overall unmatched and matched samples.^a^

Sample, subgroup, and group				Baseline, mean (SD)		Adjusted final, mean (SE; 95% CI)		Adjusted final (group A – group B), mean difference (95% CI)			
**Matched sample** **(** **n=80)**											
	**Low tertile**								2.99 (–1.80 to 7.77)		
		Group A (n=12)	49.73 (5.10)		87.33 (1.70; 83.80-90.87)						
		Group B (n=14)	N/A^b^		84.35 (1.53; 81.16-87.53)						
	**Medium tertile**								2.94 (–0.70 to 6.58)		
		Group A (n=14)	61.56 (3.15)		87.00 (1.20; 84.50-89.49)						
		Group B (n=13)	N/A		84.05 (1.28; 81.41-86.70)						
	**High tertile**								5.93 (2.17 to 9.70)		
		Group A (n=14)	72.42 (5.18)		88.14 (1.24; 85.56-90.72)						
		Group B (n=13)	N/A		82.21 (1.26; 79.60-84.82)						
**Overall unmatched sample** **(** **N=168)**											
	**Low tertile**								1.64 (–2.36 to 5.63)		
		Group A (n=33)	50.81 (6.28)		84.27 (1.29; 81.69-86.86)						
		Group B (n=23)	N/A		82.64 (1.51; 79.61-85.66)						
	**Medium tertile**								1.29 (–2.37 to 4.94)		
		Group A (n=42)	63.56 (2.60)		85.79 (0.88; 84.02-87.56)						
		Group B (n=14)	N/A		84.50 (1.57; 81.34-87.66)						
	**High tertile**								7.04 (3.84 to 10.25)		
		Group A (n=45)	75.27 (5.54)		88.41 (0.70; 87.02-89.81)						
		Group B (n=11)	N/A		81.37 (1.44; 78.49-84.25)						

^a^Matched sample models adjusted for final test case; overall unmatched sample models adjusted for final test case and baseline score. Given that the interaction analysis showed test case explained the largest proportion of variance (partial η^2^>0.43), whereas sex and major contributed minimally, these variables were not included in the models.

^b^N/A: not applicable.

## Discussion

### Overview

This study introduced LLM-VSPs as a self-directed practice tool for MHT in an authentic educational setting and evaluated their effectiveness through PSM and multidimensional robustness checks. The results indicated that the integration of LLM-VSPs effectively enhanced medical students’ MHT competency. Nevertheless, exploratory analysis suggests that this benefit may not be uniformly distributed across student cohorts with varying baseline proficiencies. Specifically, while students across all proficiency levels appeared to benefit from the practice, the high baseline students with a robust theoretical knowledge foundation tended to show a greater magnitude of improvement, whereas the gains for students with medium to low baseline levels seemed relatively limited. These observations imply that the effectiveness of LLM-driven clinical skills training tools may be influenced by learners’ prior knowledge levels, offering critical reference points for the practical deployment of such tools and the formulation of stratified teaching strategies.

### Intervention Efficacy and Robustness

Analysis of the matched samples revealed that the introduction of LLM-VSPs had a significant positive effect on enhancing medical students’ MHT skills (mean difference 3.98; *P*=.02). The positive effect remained robust (*B*=3.924; *P*<.001) even after further controlling for known confounders using a multiple linear regression model. For interventional research conducted in undergraduate teaching settings, randomized controlled trials (RCTs) represent the gold standard for causal inference. Nevertheless, random allocation of educational resources may undermine teaching homogeneity and educational equity. Therefore, this study adopted a prospective cohort design based on voluntary registration and PSM.

PSM [25,26] can simulate the randomization process of an RCT, reconstructing a new sample set with balanced baseline characteristics and thus establishing a methodological foundation for between-group comparisons. However, controlling for observed confounders is not equivalent to eliminating hidden bias [27]. A Rosenbaum bounds analysis yielded a Γ value of merely 1.21, suggesting that the conclusions remain somewhat sensitive to relatively minor hidden biases. Given the voluntary enrollment design, learning motivation is the most prominent potential unobserved confounder [28]. This raises the question of whether the outstanding performance of the intervention group was influenced by stronger learning motivation rather than the actual effect of the LLM-VSPs. Results from the ITT sensitivity analysis provide tentative supportive evidence. By retaining 9 students who voluntarily enrolled but failed to meet the effective practice standards—implying relatively lower motivation—in the intervention group, we artificially diluted the group’s motivational advantage. Instead of diminishing, the between-group difference further expanded (mean difference 3.98, 95% CI 0.55-7.40 vs mean difference 5.27, 95% CI 1.52-9.02), the effect size increased from 0.52 to 0.70, and the Rosenbaum bounds Γ value rose to 1.42. The results suggest that motivational bias likely did not inflate the intervention effect. However, we must interpret this finding cautiously. Since rematching altered the sample composition (from 40 to 33 pairs) and these 9 students might differ in attributes other than motivation, we cannot rely solely on this sensitivity analysis to definitively rule out hidden bias on the intervention effects. Nevertheless, these findings still provide supplementary support for the robustness of the LLM-VSPs intervention effects.

This finding aligns with the results of Luo et al [29] regarding virtual patients improving ophthalmology history-taking skills. LLM-VSPs provide medical students with a secure, repeatable, and accessible practice platform [30], allowing for repeated trial-and-error and consolidation of learning, thereby helping them improve their MHT competency. Furthermore, real human SPs possess greater clinical contextual authenticity and induce performance pressure; students may experience anxiety that impedes knowledge retrieval and expression. Repeated simulated interactions with LLM-VSPs can also enhance students’ self-efficacy, enabling them to remain more composed when facing real SPs [31].

### Dimensional Differences in Efficacy

A detailed analysis of the dimensional characteristics of the LLM-VSPs intervention effects revealed that the MHT score improvements facilitated by LLM-VSPs were primarily reflected in the content dimension, while the enhancement in the skills dimension was relatively limited. This finding aligns closely with the discrepancy in scoring weights between the formative assessment [32] via LLM-VSPs and the end-of-term summative assessment. Such a weighting discrepancy is not a contradiction in the study design but rather stems from the differing evaluation purposes and technical media of the two assessments. LLM-VSPs focus on helping beginners master the correct MHT content framework through a “practice-assessment-feedback-correction” cycle, with the scoring emphasizing the completeness and accuracy of the consultation content. Furthermore, constrained by the current limitations of LLM technology, LLM-VSPs struggle to simulate nonverbal communication elements during consultations, such as eye contact, gestures, and nodding. Students also exhibit challenges in conveying genuine affective reactions in their interactions with LLM-VSPs [33,34]. Consequently, the scoring and feedback primarily concentrate on the quantitative assessment of content completeness. In contrast, the end-of-term summative evaluation based on human SPs aims to comprehensively assess students’ overall clinical competence, with more balanced weights assigned to both dimensions. This outcome clearly delineates the current applicability of LLM-VSPs as a training tool for MHT skills. While LLM-VSPs predominantly fulfill the training needs in the content dimension of MHT, they fall short in providing effective practice and precise feedback for interviewing techniques, including the fluency and accessibility of language, empathy, various nonverbal communications [35], and responsiveness to patients’ emotions [36]. This finding resonates with recent research [23] conclusions regarding the application of conversational AI in medical education, which indicate that due to current technical bottlenecks in affective computing and multimodal interaction, AI systems are ill-equipped to handle the comprehensive training of communication skills. Nevertheless, with the rapid advancement of LLMs equipped with visual and auditory perception capabilities, future LLM-VSPs are expected to achieve breakthroughs in simulating nonverbal interactions [37], thereby enabling their application in more comprehensive interviewing skills practice.

### Practice Behaviors and the Cognitive Threshold

Although the preceding analysis indicated the intervention effects of LLM-VSPs on medical students, the learning mechanisms underlying whether merely providing LLM-VSPs practice tools can lead to skill improvement require further investigation. To this end, this study analyzed the association between LLM-VSP practice behaviors and final MHT scores at a deeper level. We observed a thought-provoking phenomenon: before adjusting for baseline scores, the average AI score was weakly positively correlated with the final exam score, while practice frequency and duration showed no significant association, seemingly corroborating the notion that “practice quality matters more than practice quantity.” However, after controlling for baseline scores, the significant correlation between the average AI score and the final MHT scores disappeared. Multiple linear regression further indicated that compared to LLM-VSP practice behavior indicators, baseline scores had a stronger predictive power for medical students’ final MHT competence. This suggests that in the self-directed practice mode, merely increasing practice duration or frequency may not necessarily lead to competence improvement, and AI scores appear to be strongly associated with students’ prior knowledge levels. Admittedly, these results involved multiple comparisons without strict statistical correction, merely identifying preliminary associations between practice behaviors, baseline scores, and final MHT competence.

Based on the above findings, we speculate that the self-directed use of AI-empowered practice tools may involve a “cognitive threshold.” The acquisition of MHT skills is essentially the transformation from “declarative knowledge” to “procedural skills” [1,38]. Baseline assessments mainly reflect the stock of declarative knowledge at the “remembering and understanding” cognitive tier shortly after theoretical coursework, while the final examination demands students deploy procedural skills at the “application and analysis” level in authentic interpersonal clinical encounters. The transition from “knowing what to ask” to “being able to ask fluently” not only necessitates sustained simulated practice [39,40] but, more importantly, requires the prerequisite of mastering solid declarative knowledge.

While LLM-VSPs deliver real-time quantitative feedback that precisely identifies omissions in history-taking content [32] and facilitates deliberate practice [41], learners lacking a robust foundational theoretical framework may fail to thoroughly comprehend the diagnostic reasoning underpinning AI-generated feedback. Instead, they may merely compile inquiry items in a passive manner, confining their training to superficial mechanical repetition. This hinders efficient knowledge transfer and internalization [42,43]. In contrast, students with higher baseline proficiency have already mastered the theoretical principles of history-taking and possess the cognitive capacity to thoroughly interpret AI-generated feedback [44,45]. This allows them to leverage such feedback to rectify omissions in clinical inquiry and remedy knowledge deficits within a virtuous “practice-feedback-correction” cycle. This may be the prerequisite for AI-supported deliberate practice to take effect [41]: effective practice requires not only repetition but also the correction of errors during the process.

### Differential Effects by Baseline Proficiency

To verify the above speculation, we further analyzed the potential heterogeneous effect of baseline ability on the LLM-VSP intervention. Strict interaction term tests showed that the interaction between baseline scores and group assignment did not reach statistical significance in either the matched or full samples, suggesting that LLM-VSPs appeared to demonstrate general effectiveness across different baseline levels without presenting significant statistical differences. However, given the limited sample size of this study, which might affect the statistical power of the interaction term analysis [46], we conducted an exploratory subgroup analysis to observe the data characteristics of final exam scores across different baseline levels in greater detail. Considering that PSM might lead to the loss of some high baseline samples [25], we analyzed both the full sample and the matched sample. The results revealed that while students in all baseline subgroups appeared to benefit from the use of LLM-VSPs, the high baseline subgroup showed a trend of larger between-group differences, whereas the differences in the medium and low baseline subgroups were relatively smaller. Moreover, this trend appeared more pronounced in the full sample compared to the matched sample. It should be cautiously noted that this result might be influenced by the small sample size of the control group in that subgroup (n=11). Additionally, the ICC values for several stations in this study were relatively low; such measurement error might weaken the statistical power to some extent, making it difficult to detect potential subtle skill improvements among medium and low baseline students. Nevertheless, it still aligns with our speculation: students in the high baseline group seemed to benefit more from the self-directed practice with LLM-VSPs.

Furthermore, if this trend of differential benefit holds true in larger samples, it offers a plausible explanation for why the overall effect size was only moderate. Given that students with medium or low baseline proficiency constitute the majority of the sample, their relatively limited benefit magnitudes may have diluted the larger benefits observed among the high baseline subgroup, thereby narrowing the overall mean difference. Admittedly, we must acknowledge that since the interaction term analysis did not reach statistical significance, the above heterogeneity analysis remains a trend observation, and future studies with larger samples and more rigorous designs are needed for confirmation.

### Implications for Stratified Teaching

These findings offer important reference points for the application of LLM-VSPs in *diagnostics* education. As an after-class self-directed learning tool, LLM-VSPs can enhance medical students’ MHT competency. However, maximizing the pedagogical benefits of this tool requires further reflection in teaching practice. Based on the trends observed in the aforementioned analysis, we speculate that merely increasing practice duration or frequency may not necessarily guarantee competence improvement, and the effectiveness of practice quality might be constrained by baseline ability. Therefore, paying attention to students’ baseline levels and implementing stratified interventions may help optimize teaching outcomes. For students who already possess a solid theoretical foundation, self-directed use of LLM-VSPs can facilitate the transformation of knowledge into skills. Nevertheless, for learners with medium or low baseline proficiency, structured guided support may be required to replace independent exploration to maximize the intervention benefits delivered by LLM-VSPs. Specifically, using LLM-VSPs under teacher guidance could help these students consolidate their theoretical groundwork and interpret AI feedback, thereby achieving a transition from mechanical repetition to effective practice. However, further empirical research is required to verify whether this hybrid teaching model combining instructor guidance and LLM-VSPs practice can substantially improve MHT proficiency among students with medium and low baseline ability.

Furthermore, the significance of LLM-VSPs extends beyond their immediate educational outcomes to their potential in promoting digital equity in medical education. Even in the current era of widespread AI accessibility, underresourced grassroots medical schools often struggle with limited faculty and SP resources. In these settings, educators face challenges in transforming general-purpose LLMs into effective pedagogical tools [47] and lack the capacity to undertake the laborious training required for human SPs [48]. The standardized LLM-VSPs agent developed in this study is accessible via a WeChat miniprogram. It not only eliminates development and training costs for grassroots medical institutions but also enables students at these institutions to receive high-quality history-taking training comparable to that offered by top-tier medical schools using only a smartphone and internet access. However, the accessibility of a tool does not equate to the efficiency of learning. How to assist learners of varying proficiency levels to derive equally effective practice benefits may be the key to unlocking the potential of AI teaching tools and realizing high-quality educational equity.

### Advantages and Limitations

This study validated the effectiveness of LLM-VSPs within an authentic diagnostic teaching context. It adopted PSM and multiple linear regression analysis methods to minimize confounding bias to the greatest extent, providing a methodological paradigm for causal inference in educational settings where RCTs were impractical. Simultaneously, this study extended beyond simple verification of intervention efficacy; rather, it conducted an in-depth analysis combining various behavioral data from practice with baseline levels. It preliminarily revealed the “cognitive threshold” phenomenon during the self-directed use of LLM-VSPs and identified a trend of differential benefits across different groups, providing data for the subsequent implementation of refined teaching with LLM-VSPs.

Admittedly, this study has certain limitations. First, although we used various statistical methods to control for confounding factors, Rosenbaum bounds sensitivity analysis indicated that our results remained comparatively vulnerable to unmeasured confounders; future studies require more rigorous experimental designs to draw definitive causal inferences. Second, this study was conducted as a single-center investigation with a limited sample size, and PSM also led to a reduction in sample size, which may affect the statistical power and generalizability of the research conclusions. Third, the analyses concerning the association between practice behaviors and final history-taking competency, as well as baseline heterogeneity, were exploratory in nature. The “cognitive threshold” hypothesis and the trend of “greater benefits for high baseline students” proposed in this study were primarily based on trend observations and lack robust statistical support evidence; thus, their causal mechanisms remain to be clarified. In future research, multicenter, large-sample RCTs should be conducted to generate more robust evidence in support of the findings. In addition, the LLM-VSPs agent developed herein has undergone iterative testing and refinement throughout its development phase to secure accurate automated scoring. Nevertheless, follow-up validation studies are still needed to formally establish its measurement reliability.

### Conclusion

Based on PSM analysis, this study has indicated that introducing LLM-VSPs as an after-class self-directed practice tool in *diagnostics* education may help improve medical students’ MHT competency. Exploratory analysis further suggests a trend that the educational benefits of LLM-VSPs appear more pronounced among high baseline students with firm theoretical foundations, while the improvement for medium or low baseline students is relatively limited. This phenomenon implies that during the self-directed use of LLM-driven learning tools, learners’ prior knowledge levels may constrain the efficiency of using instant AI feedback to achieve the transformation from declarative knowledge into procedural skills. Accordingly, we suggest that future teaching practices should consider students’ differences in baseline competencies and explore teaching strategies for stratified interventions when deploying LLM-VSPs. High baseline students might attempt self-directed use, whereas for medium and low baseline students, it is necessary to explore a guided teaching model combining instructor guidance and LLM-VSPs. Future research using multicenter, large-sample RCTs is needed to further verify the aforementioned differences in benefits and the effectiveness of stratified teaching strategies.

## Appendix

Multimedia Appendix 1 Development of the DoctorU teaching system for medical history-taking practice.

Multimedia Appendix 2 Scoring rubrics for virtual standardized patient cases.

Multimedia Appendix 3 The detailed participant screening flowchart.

Multimedia Appendix 4 Scoring rubrics for the final medical history-taking test.

Multimedia Appendix 5 Supplementary statistical analyses.

## Abbreviations

ICC: intraclass correlation coefficient
ITT: intention-to-treat
LLM: large language model
LLM-VSP: large language model–powered virtual standardized patient
MHT: medical history taking
OSCE: Objective Structured Clinical Examination
PSM: propensity score matching
RCT: randomized controlled trial
SMD: standardized mean difference
SP: standardized patient
VSP: virtual standardized patient

## Data Availability

The datasets generated or analyzed during this study are available from the corresponding author on reasonable request.
