# Supplementary material for: Effect of Large Language Model–Powered Virtual Standardized Patients on History-Taking Among Undergraduate Medical Students: Propensity-Matched Cohort Study
Source: JMIR Med Educ. 2026 Sep 9;12:e92486. doi: 10.2196/92486 (PMC13601875; doi:10.2196/92486)
Supplement: Supplementary file 2 [file mededu_v12i1e92486_app2.docx]

**Appendix 2**

**Scoring Rubrics for Virtual Standardized Patient Cases**

**Case1 Fever**

| **Content Structure** | **Inquiry Assessment Content** | | | **Points** |
| --- | --- | --- | --- | --- |
| Preparation and Introduction | Self-introduction | | | 1 |
|  | Explanation of duties | | | 1 |
|  | Obtaining patient cooperation | | | 1 |
| General data | 1.Patient's name | | | 0.5 |
|  | 2.Patient's gender | | | 0.5 |
|  | 3.Patient's age | | | 0.5 |
|  | 4.Patient's ethnicity | | | 0.5 |
|  | 5.Patient's native place | | | 0.5 |
|  | 6.Patient's birthplace | | | 0.5 |
|  | 7. Patient's current residence | | | 0.5 |
|  | 8.Patient's occupation | | | 0.5 |
|  | 9. Patient's marital status | | | 0.5 |
|  | 10. Patient's specific contact information | | | 0.5 |
| History of present illness | Chief complaints and characteristic | Fever | 1.Chief complaints | 4 |
|  |  |  | 2.Onset time | 4 |
|  |  |  | 3.Onset speed | 2 |
|  |  |  | 4.Causes and inducements of fever | 4 |
|  |  |  | 5.Degree of fever | 2 |
|  |  |  | 6.Progression of fever course | 6 |
|  |  |  | 7.Fever pattern | 4 |
|  |  |  | 8.Accompanying symptoms of fever | 2 |
|  |  |  | 9.Treatment for fever | 2 |
|  | Accompanying Symptoms | 1.Cough and expectoration (positive) | Nature of cough | 3 |
|  |  | 1.Headache (negative) | Confirmation of headache symptoms | 3 |
|  |  | 2.Diarrhea (negative) | Confirmation of diarrhea symptoms | 2 |
|  |  | 3.Frequent urination, urgent urination, and painful urination (negative) | Confirmation of symptoms of frequent urination, urgent urination, and painful urination | 2 |
|  | Diagnostic and Therapeutic Process | 1.Previous medical treatment institutions | | 2 |
|  |  | 2.Previous treatment measures | | 2 |
|  | Current General Condition | Appetite | | 1 |
|  |  | Defecation and urination | | 1 |
|  |  | Weight changes | | 1 |
|  |  | Mental state | | 1 |
|  |  | Sleep quality | | 1 |
|  |  | Physical strength | | 1 |
| History of past illness | 1.Chronic disease history | 1.Confirmation of other past diseases | | 2 |
|  |  | 2.Long-term medication history | | 1 |
|  | 2.Infectious disease history | 1.History of infectious diseases | | 1 |
|  | 3.Surgical and traumatic history | 1.Traumatic history | | 1 |
|  |  | 2.Surgical history | | 1 |
|  | 4.Allergy history | 1.Allergy history | | 1 |
|  | 5.Vaccination history | 1. Vaccination history | | 1 |
|  | 6.Blood transfusion history | 1.Whether there has been a blood transfusion | | 1 |
| Personal History | 1.Social experiences | 1.Long-term residence area | | 0.5 |
|  |  | 2.Epidemic source and epidemic area | | 1 |
|  | 2.Occupational working conditions | 1.Occupation and working conditions | | 1 |
|  | 3.Habits and hobbies | 1.Alcohol consumption history | | 0.5 |
|  |  | 2.Smoking history | | 1 |
|  | 4.Sexual promiscuity history | 1.History of unclean sexual intercourse | | 1 |
| Marital and Reproductive History | 1.Marital status | 1.Whether married | | 5 |
| Family History | 1.Family medical history | 1.Family genetic disease history | | 1 |
|  |  | 2.Similar family medical history | | 1 |
|  |  | 3.Parents' health conditions | | 1 |
| Inquiry Skills | Inquiry sequence | | | 5 |
|  | Communication skills | | | 5 |
|  | Complex professional vocabulary | | | 5 |
| Humanistic Care | Empathetic communication | | | 5 |

**Case2	Hemoptysis**

| **Content Structure** | **Inquiry Assessment Content** | | | **Points** |
| --- | --- | --- | --- | --- |
| Preparation and Introduction | Self-introduction | | | 1 |
|  | Explanation of duties | | | 1 |
|  | Obtaining patient cooperation | | | 1 |
| General data | 1.Patient's name | | | 0.5 |
|  | 2.Patient's gender | | | 0.5 |
|  | 3.Patient's age | | | 0.5 |
|  | 4.Patient's ethnicity | | | 0.5 |
|  | 5.Patient's native place | | | 0.5 |
|  | 6.Patient's birthplace | | | 0.5 |
|  | 7. Patient's current residence | | | 0.5 |
|  | 8.Patient's occupation | | | 0.5 |
|  | 9. Patient's marital status | | | 0.5 |
|  | 10. Patient's specific contact information | | | 0.5 |
| History of present illness | Chief complaints and characteristic | Hemoptysis | 1.Chief Complaint Symptoms | 4 |
|  |  |  | 2.Onset Time | 4 |
|  |  |  | 3.Onset Severity | 2 |
|  |  |  | 4.State During Illness Progression | 2 |
|  |  |  | 5.Amount of Hemoptysis | 2 |
|  |  |  | 6.Color of Hemoptysis | 2 |
|  |  |  | 7.Duration of Hemoptysis | 2 |
|  |  |  | 8.Frequency or Number of Hemoptysis Episodes | 2 |
|  |  |  | 9.Etiological Factors of Hemoptysis | 4 |
|  |  |  | 10.Factors Aggravating or Alleviating Hemoptysis | 2 |
|  |  |  | 11.Previous Episodes of Hemoptysis | 2 |
|  |  |  | 12.Associated Symptoms of Hemoptysis | 2 |
|  | Accompanying Symptoms | 1.Chest Pain (Positive) | 1.Confirmation of Chest Pain Symptoms | 1 |
|  |  |  | 2.Etiological Factors of Chest Pain | 1 |
|  |  |  | 3.Onset Time of Chest Pain | 0.5 |
|  |  |  | 4.Location of Chest Pain | 0.5 |
|  |  |  | 5.Severity of Chest Pain | 0.5 |
|  |  |  | 6.Duration of Chest Pain | 0.5 |
|  |  |  | 7.Nature of Chest Pain | 0.5 |
|  |  |  | 8.Factors Aggravating or Relieving Chest Pain | 1 |
|  |  |  | 9.Previous Episodes of Chest Pain | 0.5 |
|  |  | 1.Fever (Negative) | 1.Confirmation of Fever Symptoms | 1 |
|  |  | 2.Hematemesis (Negative) | 1.Confirmation of Hematemesis Symptoms | 1 |
|  |  | 3.Abdominal Pain (Negative) | 1.Confirmation of Abdominal Pain Symptoms | 1 |
|  |  | 4.Dyspnea (Negative) | 1.Confirmation of Dyspnea Symptoms | 1 |
|  | Diagnostic and Therapeutic Process | 1.Previous Examination Findings | | 0.5 |
|  |  | 2.Previous Treatment Measures | | 0.5 |
|  |  | 3.Previous Treatment Outcomes | | 0.5 |
|  |  | 4.Medication Usage | | 0.5 |
|  |  | 5.Names of Previous Medications | | 0.5 |
|  |  | 6. Efficacy of Previous Medications | | 0.5 |
|  |  | 7.Previous Diagnosis and Treatment Situations | | 0.5 |
|  |  | 8. Previous Diagnostic Results | | 0.5 |
|  | Current General Condition | Appetite | | 1 |
|  |  | Defecation and urination | | 1 |
|  |  | Weight changes | | 1 |
|  |  | Mental state | | 1 |
|  |  | Sleep quality | | 1 |
|  |  | Physical strength | | 1 |
| History of past illness | 1.Chronic disease history | 1.Confirmation of other past diseases | | 2 |
|  |  | 2.Long-term medication history | | 1 |
|  | 2.Infectious disease history | 1.History of infectious diseases | | 1 |
|  | 3.Surgical and traumatic history | 1.Traumatic history | | 1 |
|  |  | 2.Surgical history | | 1 |
|  | 4.Allergy history | 1.Allergy history | | 1 |
|  | 5.Vaccination history | 1. Vaccination history | | 1 |
|  | 6.Blood transfusion history | 1.Whether there has been a blood transfusion | | 1 |
| Personal History | 1.Social experiences | 1.Long-term residence area | | 0.5 |
|  |  | 2.Epidemic source and epidemic area | | 1 |
|  | 2.Occupational working conditions | 1.Working Conditions and Environment | | 1 |
|  | 3.Habits and hobbies | 1.Alcohol consumption history | | 1 |
|  |  | 2.Smoking history | | 1 |
|  | 4.Sexual promiscuity history | 1.History of unclean sexual intercourse | | 1 |
| Marital and Reproductive History | 1.Marital status | 1.Whether Consanguineous Marriage | | 1 |
|  |  | 2.Spouse's Physical Health Condition | | 1 |
|  |  | 3.Whether Married | | 1 |
|  |  | 4.Duration of Marriage | | 1 |
|  | 1.Children's Information | 1.Children's Information | | 1 |
| Family History | 1.Family medical history | 1.Family genetic disease history | | 1 |
|  |  | 2.Similar family medical history | | 1 |
|  |  | 3.Parents' health conditions | | 1 |
| Inquiry Skills | Inquiry sequence | | | 5 |
|  | Communication skills | | | 5 |
|  | Complex professional vocabulary | | | 5 |
| Humanistic Care | Empathetic communication | | | 5 |

**Case 3 Dyspnea**

| **Content Structure** | **Inquiry Assessment Content** | | | **Points** |
| --- | --- | --- | --- | --- |
| Preparation and Introduction | Self-introduction | | | 1 |
|  | Explanation of duties | | | 1 |
|  | Obtaining patient cooperation | | | 1 |
| General data | 1.Patient's name | | | 0.5 |
|  | 2.Patient's gender | | | 0.5 |
|  | 3.Patient's age | | | 0.5 |
|  | 4.Patient's ethnicity | | | 0.5 |
|  | 5.Patient's native place | | | 0.5 |
|  | 6.Patient's birthplace | | | 0.5 |
|  | 7. Patient's current residence | | | 0.5 |
|  | 8.Patient's occupation | | | 0.5 |
|  | 9. Patient's marital status | | | 0.5 |
|  | 10. Patient's specific contact information | | | 0.5 |
| History of present illness | Chief complaints and characteristic | Dyspnea | 1.Chief complaint symptom | 4 |
|  |  |  | 2.Onset time | 4 |
|  |  |  | 3.Onset urgency | 2 |
|  |  |  | 4.Degree of dyspnea | 4 |
|  |  |  | 5.Duration of dyspnea | 3 |
|  |  |  | 6.Frequency of dyspnea | 3 |
|  |  |  | 7.Causes and triggers of dyspnea | 4 |
|  |  |  | 8.Progression process of dyspnea | 2 |
|  |  |  | 9.Previous episodes of dyspnea | 2 |
|  |  |  | 10.Accompanying symptoms of dyspnea | 2 |
|  | Accompanying Symptoms | 1.Cough and Sputum (Positive) | 1.Confirmation of cough symptom | 1 |
|  |  |  | 2.Factors aggravating or alleviating cough | 1 |
|  |  |  | 3.Characteristics and amount of sputum | 1 |
|  |  |  | 4.Characteristics of sputum during expectoration | 1 |
|  |  | 2.Edema (Positive) | 1.Confirmation of edema symptom | 1 |
|  |  |  | 2.Onset time of edema | 0.5 |
|  |  |  | 3.Degree of edema | 0.5 |
|  |  |  | 4.Causes and triggers of edema | 0.5 |
|  |  |  | 5.Previous episodes of edema | 0.5 |
|  |  | 1.Chest Pain (Negative) | 1.Confirmation of chest pain symptom | 1 |
|  |  | 2.Fever (Negative) | 1.Confirmation of fever symptom | 1 |
|  |  | 3.Hemoptysis (Negative) | 1.Confirmation of hemoptysis symptom | 1 |
|  | Diagnostic and Therapeutic Process | 1.Previous examination results | | 0.5 |
|  |  | 2.Previous treatment measures | | 0.5 |
|  |  | 3.Previous medication names | | 0.5 |
|  |  | 4.Previous medication duration | | 0.5 |
|  |  | 5.Previous medication efficacy | | 0.5 |
|  |  | 6.Previous examination findings | | 0.5 |
|  |  | 7.Previous medical records and examination results | | 0.5 |
|  |  | 8.Previous diagnosis results | | 0.5 |
|  | Current General Condition | Appetite | | 1 |
|  |  | Defecation and urination | | 1 |
|  |  | Weight changes | | 1 |
|  |  | Mental state | | 1 |
|  |  | Sleep quality | | 1 |
|  |  | Physical strength | | 1 |
| History of past illness | 1.Chronic disease history | 1.Confirmation of previous other diseases | | 1 |
|  |  | 2.Previous treatment for chronic diseases | | 1 |
|  |  | 3.Previous tuberculosis condition | | 1 |
|  |  | 4.Long-term medication history | | 1 |
|  | 2.Surgical and traumatic history | 1.Traumatic history | | 1 |
|  |  | 2.Surgical history | | 1 |
|  | 3.Allergy history | 1.Allergy history | | 1 |
|  | 4.Vaccination history | 1.Vaccination history | | 1 |
|  | 5.Blood transfusion history | 1.Whether there has been a blood transfusion | | 1 |
| Personal History | 1.Social experiences | 1.Long-term residence area | | 0.5 |
|  |  | 2.Epidemic source and epidemic area | | 0.5 |
|  | 2.Occupational working conditions | 1.Occupational type | | 0.5 |
|  |  | 2.Working conditions and environment | | 1 |
|  | 3.Habits and hobbies | 1.Alcohol consumption history | | 0.5 |
|  |  | 2.Smoking history | | 0.5 |
|  |  | 3.Amount of smoking | | 0.5 |
|  | 4.Sexual promiscuity history | 1.History of unclean sexual intercourse | | 1 |
| Marital and Reproductive History | 1.Marital status | 1.Previous marital status | | 0.5 |
|  |  | 2.Whether it is a consanguineous marriage | | 1 |
|  |  | 3.Health condition of the spouse | | 1 |
|  |  | 4.Whether married | | 0.5 |
|  |  | 5.Duration of marriage | | 1 |
|  | 2.Children's situation | 1.Children's situation | | 1 |
| Family History | 1.Family medical history | 1.Family genetic disease history | | 1 |
|  |  | 2.Similar family medical history | | 1 |
|  |  | 3.Parents' health conditions | | 1 |
| Inquiry Skills | Inquiry sequence | | | 5 |
|  | Communication skills | | | 5 |
|  | Complex professional vocabulary | | | 5 |
| Humanistic Care | Empathetic communication | | | 5 |

**Case 4 Cough and Sputum**

| **Content Structure** | **Inquiry Assessment Content** | | | **Points** |
| --- | --- | --- | --- | --- |
| Preparation and Introduction | Self-introduction | | | 1 |
|  | Explanation of duties | | | 1 |
|  | Obtaining patient cooperation | | | 1 |
| General data | 1.Patient's name | | | 0.5 |
|  | 2.Patient's gender | | | 0.5 |
|  | 3.Patient's age | | | 0.5 |
|  | 4.Patient's ethnicity | | | 0.5 |
|  | 5.Patient's native place | | | 0.5 |
|  | 6.Patient's birthplace | | | 0.5 |
|  | 7. Patient's current residence | | | 0.5 |
|  | 8.Patient's occupation | | | 0.5 |
|  | 9. Patient's marital status | | | 0.5 |
|  | 10. Patient's specific contact information | | | 0.5 |
| History of present illness | Chief complaints and characteristic | Cough and sputum | 1.Chief complaint symptom | 4 |
|  |  |  | 2.Onset time | 4 |
|  |  |  | 3.Onset urgency | 2 |
|  |  |  | 4.Cough interval pattern | 1 |
|  |  |  | 5. Causes and inducements of cough | 4 |
|  |  |  | 6.Factors aggravating or alleviating cough | 2 |
|  |  |  | 7.Nature of cough | 1 |
|  |  |  | 8.Sputum characteristics and volume | 2 |
|  |  |  | 9.Previous episodes of cough | 2 |
|  |  |  | 10.Associated symptoms of cough | 2 |
|  |  |  | 11.Duration of cough | 2 |
|  |  |  | 12.Sputum Characteristics during Expectoration | 2 |
|  |  |  | 13. Severity of cough onset | 2 |
|  | Accompanying Symptoms | 1.Dyspnea (positive) | 1.Confirmation of dyspnea symptoms | 1 |
|  |  |  | 2.Factors alleviating or aggravating dyspnea | 0.5 |
|  |  |  | 3.Causes and inducements of dyspnea condition | 0.5 |
|  |  |  | 4.Previous episodes of dyspnea | 0.5 |
|  |  | 2.Fever (Positive) | 1.Confirmation of fever symptoms | 1 |
|  |  |  | 2.Causes and inducements of fever | 0.5 |
|  |  |  | 3.Onset time of fever | 0.5 |
|  |  |  | 4.Degree of fever | 0.5 |
|  |  |  | 5.Factors aggravating or alleviating fever | 1 |
|  |  |  | 6.Previous episodes of fever | 0.5 |
|  |  |  | 7.Treatment for fever | 0.5 |
|  |  | 1.Chest pain (Negative) | 1.Confirmation of chest pain symptoms | 1 |
|  |  | 2.Hemoptysis (Negative) | 1.Confirmation of hemoptysis symptoms | 1 |
|  |  | 3.Edema (Negative) | 1.Confirmation of edema symptoms | 1 |
|  | Diagnostic and Therapeutic Process | 1.Previous medical treatment unit | | 0.5 |
|  |  | 2.Effectiveness of previous treatment | | 0.5 |
|  |  | 3.Names of previous medications | | 0.5 |
|  |  | 4.Efficacy of previous medications | | 0.5 |
|  |  | 5.Previous examination results | | 0.5 |
|  |  | 6.Previous medical records and examination results | | 0.5 |
|  |  | 7. Previous diagnosis results | | 0.5 |
|  |  | 8.Follow-up examination after treatment | | 0.5 |
|  | Current General Condition | Appetite | | 1 |
|  |  | Defecation and urination | | 1 |
|  |  | Weight changes | | 1 |
|  |  | Mental state | | 1 |
|  |  | Sleep quality | | 1 |
|  |  | Physical strength | | 1 |
| History of past illness | 1.History of Infectious Diseases | 1.History of infectious diseases | | 2 |
|  | 2.History of Chronic Diseases | 1.Confirmation of other previous diseases | | 1 |
|  |  | 2.Previous emphysema condition | | 1 |
|  | 3.Surgical and traumatic history | 1.Traumatic history | | 1 |
|  |  | 2.Surgical history | | 1 |
|  | 4.Allergy history | 1.Allergy history | | 1 |
|  | 5.Vaccination history | 1.Vaccination history | | 1 |
|  | 6.Blood transfusion history | 1.Whether there has been a blood transfusion | | 1 |
| Personal History | 1.Social experiences | 1.Long-term residence area | | 0.5 |
|  |  | 2.Epidemic source and epidemic area | | 0.5 |
|  | 2.Occupational working conditions | 1.Working conditions and environment | | 1 |
|  | 3.Habits and hobbies | 1.Alcohol consumption history | | 1 |
|  |  | 2.Smoking history | | 1 |
|  | 4.Sexual promiscuity history | 1.History of unclean sexual intercourse | | 1 |
| Marital and Reproductive History | 1.Spouse's Health Status | 1.Spouse's Health Status | | 0.5 |
|  | 2.Menstrual History | 1.Age of menarche | | 0.5 |
|  |  | 2. Time of last menstruation | | 0.5 |
|  |  | 3.Whether menopause has occurred | | 0.5 |
|  |  | 4.Age of menopause | | 0.5 |
|  |  | 5. Whether there is vaginal bleeding after menopause | | 0.5 |
|  | 3.Childbearing History | 1.Number of children | | 0.5 |
|  |  | 2.Children's health status | | 0.5 |
|  | 4.Marital Status | 1.Whether married | | 0.5 |
|  |  | 2.Duration of marriage | | 0.5 |
| Family History | 1.Family medical history | 1.Family genetic disease history | | 1 |
|  |  | 2.Similar family medical history | | 1 |
|  |  | 3.Parents' health conditions | | 1 |
| Inquiry Skills | Inquiry sequence | | | 5 |
|  | Communication skills | | | 5 |
|  | Complex professional vocabulary | | | 5 |
| Humanistic Care | Empathetic communication | | | 5 |

**Case 5 Palpitations**

| **Content Structure** | **Inquiry Assessment Content** | | | **Points** |
| --- | --- | --- | --- | --- |
| Preparation and Introduction | Self-introduction | | | 1 |
|  | Explanation of duties | | | 1 |
|  | Obtaining patient cooperation | | | 1 |
| General data | 1.Patient's name | | | 0.5 |
|  | 2.Patient's gender | | | 0.5 |
|  | 3.Patient's age | | | 0.5 |
|  | 4.Patient's ethnicity | | | 0.5 |
|  | 5.Patient's native place | | | 0.5 |
|  | 6.Patient's birthplace | | | 0.5 |
|  | 7. Patient's current residence | | | 0.5 |
|  | 8.Patient's occupation | | | 0.5 |
|  | 9. Patient's marital status | | | 0.5 |
|  | 10. Patient's specific contact information | | | 0.5 |
| History of present illness | Chief complaints and characteristic | Palpitations | 1.Chief complaint symptom | 4 |
|  |  |  | 2.Onset time | 4 |
|  |  |  | 3.Onset urgency | 2 |
|  |  |  | 4.State during the course of the illness | 2 |
|  |  |  | 5.Intensity of palpitations | 3 |
|  |  |  | 6.Duration of palpitations | 2 |
|  |  |  | 7.Frequency of palpitations | 2 |
|  |  |  | 8.Causes and inducements of palpitations | 4 |
|  |  |  | 9. Factors aggravating or alleviating palpitations | 2 |
|  |  |  | 10.Previous episodes of palpitations | 2 |
|  |  |  | 11.Associated symptoms of palpitations | 3 |
|  | Accompanying Symptoms | 1.Dyspnea (positive) | 1.Confirmation of dyspnea symptoms | 1 |
|  |  |  | 2.Onset time of dyspnea | 1 |
|  |  |  | 3.Degree of dyspnea | 1 |
|  |  |  | 4.Duration of dyspnea | 1 |
|  |  | 1.Chest pain (negative) | 1.Confirmation of chest pain symptoms | 1 |
|  |  | 2.Fever (negative) | 1.Confirmation of fever symptom | 1 |
|  |  | 3.Cough and sputum (negative) | 1. Confirmation of cough symptom | 1 |
|  |  | 4.Nausea and vomiting (negative) | 1.Confirmation of nausea and vomiting symptom | 1 |
|  |  | 5.Syncope (negative) | 1. Confirmation of syncope symptom | 1 |
|  |  | 6.Chest tightness (negative) | 1. Confirmation of chest tightness symptom | 1 |
|  | Diagnostic and Therapeutic Process | 1.Previous medical treatment unit | | 0.5 |
|  |  | 2.Previous examination situation | | 1 |
|  |  | 3. Previous treatment measures | | 1 |
|  |  | 4.Medication situation | | 0.5 |
|  |  | 5.Previous examination results | | 0.5 |
|  |  | 6.Previous diagnosis results | | 0.5 |
|  | Current General Condition | Appetite | | 1 |
|  |  | Defecation and urination | | 1 |
|  |  | Weight changes | | 1 |
|  |  | Mental state | | 1 |
|  |  | Sleep quality | | 1 |
|  |  | Physical strength | | 1 |
| History of past illness | 1.Chronic disease history | 1. Confirmation of other previous diseases | | 1 |
|  |  | 2.Previous treatment situation for chronic diseases | | 0.5 |
|  |  | 3.Previous control situation of chronic diseases | | 0.5 |
|  |  | 4. Long-term medication history | | 1 |
|  | 2.Infectious disease history | 1.Exposure to infectious diseases history | | 0.5 |
|  |  | 2.Contracting infectious diseases history | | 0.5 |
|  | 3.Surgical and traumatic history | 1.Traumatic history | | 1 |
|  |  | 2.Surgical history | | 1 |
|  | 4.Allergy history | 1.Allergy history | | 1 |
|  | 5.Vaccination history | 1.Vaccination history | | 1 |
|  | 6.Blood transfusion history | 1.Whether there has been a blood transfusion | | 1 |
| Personal History | 1.Social experiences | 1.Long-term residence area | | 0.5 |
|  |  | 2.Epidemic source and epidemic area | | 0.5 |
|  | 2.Family situation | 1.Marital status | | 1 |
|  | 3.Habits and hobbies | 1.Alcohol consumption history | | 0.5 |
|  |  | 2.Smoking history | | 0.5 |
|  | 4.Sexual promiscuity history | 1.History of unclean sexual intercourse | | 1 |
|  | 5.Occupational working conditions | 1.Occupation and working conditions | | 1 |
| Marital and Reproductive History | 1.Marital status | 1.Age at marriage | | 0.5 |
|  |  | 2.Spouse's physical health status | | 0.5 |
|  | 2.Childbearing history | 1.Number of children | | 0.5 |
|  |  | 2.Children's health status | | 0.5 |
|  | 3.Menstrual history | 1.Age at menarche | | 0.5 |
|  |  | 2.Regularity of menstruation | | 0.5 |
|  |  | 3.Menstrual cycle | | 0.5 |
|  |  | 4.Duration of menstruation | | 0.5 |
|  |  | 5.Age at menopause | | 0.5 |
|  |  | 6.Postmenopausal vaginal bleeding | | 0.5 |
| Family History | 1.Family medical history | 1.Family genetic disease history | | 1 |
|  |  | 2.Similar family medical history | | 1 |
|  |  | 3.Parents' health conditions | | 1 |
| Inquiry Skills | Inquiry sequence | | | 5 |
|  | Communication skills | | | 5 |
|  | Complex professional vocabulary | | | 5 |
| Humanistic Care | Empathetic communication | | | 5 |

**Case 6 Cyanosis**

| **Content Structure** | **Inquiry Assessment Content** | | | **Points** |
| --- | --- | --- | --- | --- |
| Preparation and Introduction | Self-introduction | | | 1 |
|  | Explanation of duties | | | 1 |
|  | Obtaining patient cooperation | | | 1 |
| General data | 1.Patient's name | | | 0.5 |
|  | 2.Patient's gender | | | 0.5 |
|  | 3.Patient's age | | | 0.5 |
|  | 4.Patient's ethnicity | | | 0.5 |
|  | 5.Patient's native place | | | 0.5 |
|  | 6.Patient's birthplace | | | 0.5 |
|  | 7. Patient's current residence | | | 0.5 |
|  | 8.Patient's occupation | | | 0.5 |
|  | 9. Patient's marital status | | | 0.5 |
|  | 10. Patient's specific contact information | | | 0.5 |
| History of present illness | Chief complaints and characteristic | Cyanosis | 1.Chief complaint symptom | 4 |
|  |  |  | 2.Onset time | 4 |
|  |  |  | 3.Onset speed | 2 |
|  |  |  | 4.Degree of cyanosis | 4 |
|  |  |  | 5.Duration of cyanosis | 3 |
|  |  |  | 6.Frequency of cyanosis | 3 |
|  |  |  | 7.Inducements of cyanosis | 4 |
|  |  |  | 8.Factors aggravating or alleviating cyanosis symptoms | 2 |
|  |  |  | 9.Development and evolution process of cyanosis \| | 2 |
|  |  |  | 10. Associated symptoms of cyanosis | 2 |
|  | Accompanying symptoms | 1.Dyspnea (positive) | 1. Confirmation of dyspnea symptoms | 0.5 |
|  |  |  | 2. Onset time of dyspnea | 0.5 |
|  |  |  | 3. Degree of dyspnea | 0.5 |
|  |  |  | 4.Duration of dyspnea | 0.5 |
|  |  |  | 5.Frequency of dyspnea | 0.5 |
|  |  |  | 6. Causes and inducements of dyspnea | 0.5 |
|  |  | 2.Abdominal distension (positive) | 1.Onset situation and duration of abdominal distension | 0.5 |
|  |  |  | 2.Location of abdominal distension symptoms | 0.5 |
|  |  |  | 3.Degree of abdominal distension symptoms | 0.5 |
|  |  |  | 4.Causes and inducements of abdominal distension condition | 0.5 |
|  |  | 3.Edema (positive) | 1.Onset time of edema | 0.5 |
|  |  |  | 2.Location of edema | 0.5 |
|  |  |  | 3.Degree of edema | 0.5 |
|  |  |  | 4.Causes and inducements of edema | 0.5 |
|  |  | 1.Chest Pain (negative) | 1.Confirmation of chest pain symptom | 0.5 |
|  |  | 2.Fever (negative) | 1.Confirmation of fever symptom | 0.5 |
|  |  | 3.Cough and sputum (negative) | 1.Confirmation of cough symptom | 0.5 |
|  |  | 4.Hemoptysis (negative) | 1.Confirmation of hemoptysis symptom | 0.5 |
|  |  | 5.Nausea and vomiting (negative) | 1.Confirmation of nausea and vomiting symptom | 0.5 |
|  |  | 6.Syncope (negative) | 1.Confirmation of syncope symptom | 0.5 |
|  | Diagnostic and therapeutic process | 1.Previous medical treatment unit | | 1 |
|  |  | 2.Previous examination situation | | 1 |
|  |  | 3.Previous treatment measures | | 1 |
|  |  | 4.Medication situation | | 1 |
|  | Current general condition | Appetite | | 1 |
|  |  | Defecation and urination | | 1 |
|  |  | Weight changes | | 1 |
|  |  | Mental state | | 1 |
|  |  | Sleep quality | | 1 |
|  |  | Physical strength | | 1 |
| History of past illness | 1.Chronic disease history | 1.Confirmation of other previous diseases | | 1 |
|  |  | 2.Previous treatment situation for chronic diseases | | 1 |
|  |  | 3.Long-term medication history | | 1 |
|  | 2.Infectious disease History | 1.Exposure to infectious diseases history | | 0.5 |
|  |  | 2.Infectious diseases history | | 0.5 |
|  | 3.Surgical and traumatic history | 1.Traumatic history | | 1 |
|  |  | 2.Surgical history | | 1 |
|  | 4.Allergy history | 1.Allergy history | | 1 |
|  | 5.Vaccination history | 1.Vaccination history | | 1 |
|  | 6.Blood transfusion history | 1.Whether there has been a blood transfusion | | 1 |
| Personal History | 1.Social experiences | 1.Long-term residence area | | 0.5 |
|  |  | 2.Epidemic source and endemic areas | | 0.5 |
|  | 2.Family situation | 1.Family relationships | | 0.5 |
|  |  | 2.Marital status | | 0.5 |
|  | 3.Occupational working conditions | 1.Occupation & Type of Work | | 0.5 |
|  |  | 2.Working Conditions & Environment | | 0.5 |
|  | 4.Habits and hobbies | 1.Alcohol consumption history | | 0.5 |
|  |  | 2.Smoking history | | 0.5 |
|  | 5.Sexual promiscuity history | 1.History of unclean sexual intercourse | | 1 |
| Marital and Reproductive History | 1.Marital status | 1.Whether married | | 2.5 |
|  | 2.Children's Situation | 1.Information about children | | 2.5 |
| Family History | 1.Family medical history | 1.Family genetic disease history | | 1 |
|  |  | 2.Similar family medical history | | 1 |
|  |  | 3.Parents' health conditions | | 1 |
| Inquiry Skills | Inquiry sequence | | | 5 |
|  | Communication skills | | | 5 |
|  | Complex professional vocabulary | | | 5 |
| Humanistic Care | Empathetic communication | | | 5 |

**Case 7 Abdominal Pain**

| **Content Structure** | **Inquiry Assessment Content** | | | **Points** |
| --- | --- | --- | --- | --- |
| Preparation and Introduction | Self-introduction | | | 1 |
|  | Explanation of duties | | | 1 |
|  | Obtaining patient cooperation | | | 1 |
| General data | 1.Patient's name | | | 0.5 |
|  | 2.Patient's gender | | | 0.5 |
|  | 3.Patient's age | | | 0.5 |
|  | 4.Patient's ethnicity | | | 0.5 |
|  | 5.Patient's native place | | | 0.5 |
|  | 6.Patient's birthplace | | | 0.5 |
|  | 7. Patient's current residence | | | 0.5 |
|  | 8.Patient's occupation | | | 0.5 |
|  | 9. Patient's marital status | | | 0.5 |
|  | 10. Patient's specific contact information | | | 0.5 |
| History of present illness | Chief complaints and characteristic | Abdominal Pain | 1.Chief complaint symptom | 4 |
|  |  |  | 2.Onset time | 4 |
|  |  |  | 3.Onset speed | 2 |
|  |  |  | 4.Location of abdominal pain | 4 |
|  |  |  | 5.Nature of abdominal pain | 2 |
|  |  |  | 6.Degree of abdominal pain | 2 |
|  |  |  | 7.Duration of abdominal pain episodes | 2 |
|  |  |  | 8.Causes and inducements of abdominal pain | 4 |
|  |  |  | 9.Factors aggravating or alleviating abdominal pain | 2 |
|  |  |  | 10.Course and development of abdominal pain | 2 |
|  |  |  | 11.Radiation of abdominal pain symptoms | 2 |
|  | Accompanying Symptoms | 1.Belching (Positive) | 1.Confirmation of belching symptoms | 2 |
|  |  |  | 2.Associated symptoms with belching | 1 |
|  |  | 1.Fever (Negative) | 1.Confirmation of fever symptoms | 1 |
|  |  | 2.Nausea and Vomiting (Negative) | 1.Confirmation of nausea and vomiting symptoms | 1 |
|  |  | 3.Diarrhea (Negative) | 1.Confirmation of diarrhea symptoms | 0.5 |
|  |  | 4.Fatigue (Negative) | 1.Confirmation of fatigue symptoms | 0.5 |
|  |  | 5.Dizziness (Negative) | 1.Confirmation of dizziness symptoms | 1 |
|  |  | 6.Palpitations (Negative) | 1.Confirmation of palpitations symptoms | 0.5 |
|  |  | 7.Cold Sweats (Negative) | 1.Confirmation of cold sweat symptoms | 0.5 |
|  |  | 8.Chills (Negative) | 1.Confirmation of chills symptoms | 0.5 |
|  |  | 9.Thirst (Negative) | 1.Confirmation of thirst symptoms | 1 |
|  |  | 10.Jaundice (Negative) | 1.Confirmation of jaundice symptoms | 0.5 |
|  | Diagnostic and Therapeutic Process | Previous medical treatment unit | | 2 |
|  |  | Names of previously used medications | | 2 |
|  | Current General Condition | Appetite | | 1 |
|  |  | Defecation and urination | | 1 |
|  |  | Weight changes | | 1 |
|  |  | Mental state | | 1 |
|  |  | Sleep quality | | 1 |
|  |  | Physical strength | | 1 |
| History of past illness | 1.Chronic disease history | 1.Confirmation of other previous diseases | | 1 |
|  |  | 2.Control situation of previous chronic diseases | | 1 |
|  |  | 3.Long-term medication history | | 1 |
|  | 2.Infectious Disease History | 1.Exposure to infectious diseases history | | 0.5 |
|  |  | 2.Infectious diseases history | | 0.5 |
|  | 3.Surgical and traumatic history | 1.Traumatic history | | 1 |
|  |  | 2.Surgical history | | 1 |
|  | 4.Allergy history | 1.Allergy history | | 1 |
|  | 5.Vaccination history | 1.Vaccination history | | 1 |
|  | 6.Blood transfusion history | 1.Whether there has been a blood transfusion | | 1 |
| Personal History | 1.Social experiences | 1.Long-term residence area | | 0.5 |
|  |  | 2.Epidemic source and epidemic area | | 0.5 |
|  | 2.Occupational working conditions | 1.Occupational type | | 0.5 |
|  | 3.Habits and hobbies | 1.Alcohol consumption history | | 1 |
|  |  | 2.Smoking history | | 0.5 |
|  |  | 3.Dietary habits | | 1 |
|  | 4.Sexual promiscuity history | 1.History of unclean sexual intercourse | | 0.5 |
|  |  | 2.History of sexually transmitted diseases | | 0.5 |
| Marital and Reproductive History | 1.Marital status | 1.Age at marriage | | 1 |
|  |  | 2.Physical health status of spouse | | 1 |
|  |  | 3.Whether married | | 1 |
|  | 2.Children's situation | 1.Information about children | | 1 |
|  |  | 2.Health of children | | 1 |
| Family History | 1.Family medical history | 1.Family genetic disease history | | 1 |
|  |  | 2.Similar family medical history | | 1 |
|  |  | 3.Parents' health conditions | | 1 |
| Inquiry Skills | Inquiry sequence | | | 5 |
|  | Communication skills | | | 5 |
|  | Complex professional vocabulary | | | 5 |
| Humanistic Care | Empathetic communication | | | 5 |

**Case8 Hematemesis**

| **Content Structure** | **Inquiry Assessment Content** | | | **Points** |
| --- | --- | --- | --- | --- |
| Preparation and Introduction | Self-introduction | | | 1 |
|  | Explanation of duties | | | 1 |
|  | Obtaining patient cooperation | | | 1 |
| General data | 1.Patient's name | | | 0.5 |
|  | 2.Patient's gender | | | 0.5 |
|  | 3.Patient's age | | | 0.5 |
|  | 4.Patient's ethnicity | | | 0.5 |
|  | 5.Patient's native place | | | 0.5 |
|  | 6.Patient's birthplace | | | 0.5 |
|  | 7. Patient's current residence | | | 0.5 |
|  | 8.Patient's occupation | | | 0.5 |
|  | 9. Patient's marital status | | | 0.5 |
|  | 10. Patient's specific contact information | | | 0.5 |
| History of present illness | Chief complaints and characteristic | Hematemesis | 1.Chief complaint symptom | 4 |
|  |  |  | 2.Onset time | 4 |
|  |  |  | 3.Onset speed | 2 |
|  |  |  | 4.Onset time of hematemesis | 2 |
|  |  |  | 5.Volume of hematemesis | 2 |
|  |  |  | 6.Color and characteristics of hematemesis | 2 |
|  |  |  | 7.Frequency of hematemesis | 2 |
|  |  |  | 8.Causes and inducements of hematemesis | 4 |
|  |  |  | 9.Factors aggravating or alleviating hematemesis | 4 |
|  |  |  | 10.Associated symptoms of hematemesis | 4 |
|  | Accompanying Symptoms | 1.Fatigue (Positive) | 1.Confirmation of fatigue symptoms | 1 |
|  |  | 2.Palpitations (Positive) | 1.Confirmation of palpitations symptoms | 1 |
|  |  | 3.Melena (Positive) | 1.Confirmation of melena symptoms | 1 |
|  |  |  | 1.Characteristics or frequency of melena onset | 1 |
|  |  | 1.Shortness of Breath (Negative) | 1.Confirmation of shortness of breath symptoms | 0.5 |
|  |  | 2.Abdominal Pain (Negative) | 1.Confirmation of abdominal pain symptoms | 0.5 |
|  |  | 3.Blurred Vision (Negative) | 1.Confirmation of blurred vision symptoms | 0.5 |
|  |  | 4.Cold Sweats (Negative) | 1.Confirmation of cold sweat symptoms | 0.5 |
|  |  | 5.Fever (Negative) | 1.Confirmation of fever symptoms | 0.5 |
|  |  | 6.Syncope (Negative) | 1.Confirmation of syncope symptoms | 0.5 |
|  |  | 7.Skin Itching (Negative) | 1.Confirmation of skin itching symptoms | 0.5 |
|  |  | 8.Thirst (Negative) | 1. Confirmation of thirst symptoms | 0.5 |
|  |  | 9.Skin Ecchymosis and Petechiae (Negative) | 1.Confirmation of skin ecchymosis and petechiae symptoms | 0.5 |
|  |  | 10.Dizziness (Negative) | 1.Confirmation of dizziness symptoms | 0.5 |
|  |  | 11.Jaundice (Negative) | 1.Confirmation of jaundice symptoms | 0.5 |
|  |  | 12.Hepatosplenomegaly (Negative) | 1.Confirmation of hepatosplenomegaly symptoms | 0.5 |
|  | Diagnostic and Therapeutic Process | Previous medical treatment unit | | 2 |
|  |  | Names of previously used medications | | 1 |
|  |  | Time of previous medication administration | | 1 |
|  | Current General Condition | Appetite | | 1 |
|  |  | Defecation and urination | | 1 |
|  |  | Weight changes | | 1 |
|  |  | Mental state | | 1 |
|  |  | Sleep quality | | 1 |
|  |  | Physical strength | | 1 |
| History of past illness | 1.Chronic disease history | 1.Confirmation of other previous diseases | | 2 |
|  |  | 2.Long-term medication history | | 1 |
|  | 2.History of Infectious Diseases | 1.History of exposure to infectious diseases | | 0.5 |
|  |  | 2.History of infectious diseases | | 0.5 |
|  | 3.Surgical and traumatic history | 1.Traumatic history | | 1 |
|  |  | 2.Surgical history | | 1 |
|  | 4.Allergy history | 1.Allergy history | | 1 |
|  | 5.Vaccination history | 1.Vaccination history | | 1 |
|  | 6.Blood transfusion history | 1.Whether there has been a blood transfusion | | 1 |
| Personal History | 1.Social experiences | 1.Long-term residence area | | 0.5 |
|  |  | 2.Epidemic focus and endemic area | | 0.5 |
|  | 2.Occupational working conditions | 1.Occupation and working conditions | | 1 |
|  | 3.Habits and hobbies | 1.Alcohol consumption history | | 1 |
|  |  | 2.Smoking history | | 0.5 |
|  |  | 3.Smoking amount | | 0.5 |
|  | 4.Sexual promiscuity history | 1.History of unclean sexual intercourse | | 0.5 |
|  |  | 2.History of sexually transmitted diseases | | 0.5 |
| Marital and Reproductive History | 1.Marital status | 1.Age at marriage | | 1 |
|  |  | 2.Whether married | | 1 |
|  | 2.Spouse's health status | 1.Spouse's health status | | 1 |
|  | 3.Children's information | 1.Number of children | | 1 |
|  |  | 2.Children's health | | 1 |
| Family History | 1.Family medical history | 1.Family genetic disease history | | 1 |
|  |  | 2.Similar family medical history | | 1 |
|  |  | 3.Parents' health conditions | | 1 |
| Inquiry Skills | Inquiry sequence | | | 5 |
|  | Communication skills | | | 5 |
|  | Complex professional vocabulary | | | 5 |
| Humanistic Care | Empathetic communication | | | 5 |

**Case9 **Polyuria****

| **Content Structure** | **Inquiry Assessment Content** | | | **Points** |
| --- | --- | --- | --- | --- |
| Preparation and Introduction | Self-introduction | | | 1 |
|  | Explanation of duties | | | 1 |
|  | Obtaining patient cooperation | | | 1 |
| General data | 1.Patient's name | | | 0.5 |
|  | 2.Patient's gender | | | 0.5 |
|  | 3.Patient's age | | | 0.5 |
|  | 4.Patient's ethnicity | | | 0.5 |
|  | 5.Patient's native place | | | 0.5 |
|  | 6.Patient's birthplace | | | 0.5 |
|  | 7. Patient's current residence | | | 0.5 |
|  | 8.Patient's occupation | | | 0.5 |
|  | 9. Patient's marital status | | | 0.5 |
|  | 10. Patient's specific contact information | | | 0.5 |
| History of present illness | Chief complaints and characteristic | **Polyuria** | 1.Chief complaint symptom | 4 |
|  |  |  | 2.Onset time | 4 |
|  |  |  | 3.Onset urgency | 2 |
|  |  |  | 4.Urine volume during polyuria | 4 |
|  |  |  | 5.Color and characteristics of urine during polyuria | 3 |
|  |  |  | 6.Urination frequency and number of times during polyuria | 3 |
|  |  |  | 7.Inducing factors of polyuria | 4 |
|  |  |  | 8.Aggravating or relieving factors of polyuria symptoms | 2 |
|  |  |  | 9.Previous episodes of polyuria | 2 |
|  |  |  | 10.Associated symptoms of polyuria | 2 |
|  | Accompanying Symptoms | **1.Thirst (Positive)** | 1.Confirmation of **thirst** symptom | 2 |
|  |  |  | 2.Onset and duration of **thirst** | 1 |
|  |  |  | 3.Severity of **thirst** symptom | 1 |
|  |  |  | 4.Aggravating or relieving factors of **thirst** symptom | 1 |
|  |  | 1.E**maciation (Negative)** | 1.Confirmation of **emaciation** symptom | 2 |
|  |  | **2.Frequent Urination, Urgency, and Dysuria (Negative)** | 1.Confirmation of frequent urination, urgency, and dysuria symptoms | 2 |
|  |  | **3.Hematuria (Negative)** | 1.Confirmation of hematuria symptom | 1 |
|  | **Diagnostic and Therapeutic Process** | Previous examination results | | 2 |
|  |  | Previous treatment measures | | 2 |
|  | Current General Condition | Appetite | | 1 |
|  |  | Defecation and urination | | 1 |
|  |  | Weight changes | | 1 |
|  |  | Mental state | | 1 |
|  |  | Sleep quality | | 1 |
|  |  | Physical strength | | 1 |
| History of past illness | 1.Chronic disease history | 1.Confirmation of other previous diseases | | 2 |
|  |  | 2.Long-term medication history | | 1 |
|  | 2.Infectious disease history | 1.History of infectious diseases | | 1 |
|  | 3.Surgical and traumatic history | 1.Traumatic history | | 1 |
|  |  | 2.Surgical history | | 1 |
|  | 4.Allergy history | 1.Allergy history | | 1 |
|  | 5.Vaccination history | 1.Vaccination history | | 1 |
|  | 6.Blood transfusion history | 1.Whether there has been a blood transfusion | | 1 |
| Personal History | 1.Social experiences | 1.Long-term residence area | | 0.5 |
|  |  | 2.Epidemic focus and endemic area | | 0.5 |
|  | 2.Occupational working conditions | 1.Occupational type | | 0.5 |
|  |  | 2.Occupation and working conditions | | 0.5 |
|  | 3.Habits and hobbies | 1.Alcohol consumption history | | 1 |
|  |  | 2.Smoking history | | 0.5 |
|  |  | 3.Smoking start time | | 0.3 |
|  |  | 4.Smoking amount | | 0.2 |
|  | 4.Sexual promiscuity history | 1.History of unclean sexual intercourse | | 1 |
| Marital and Reproductive History | 1.Marital status | 1.Age at marriage | | 1 |
|  |  | 2.Whether married | | 1 |
|  | 2.Spouse's health status | 1.Spouse's health status | | 1 |
|  | 3.Children's information | 1.Children's information | | 1 |
|  |  | 2.Children's health | | 1 |
| Family History | 1.Family medical history | 1.Family genetic disease history | | 1 |
|  |  | 2.Similar family medical history | | 1 |
|  |  | 3.Parents' health conditions | | 1 |
| Inquiry Skills | Inquiry sequence | | | 5 |
|  | Communication skills | | | 5 |
|  | Complex professional vocabulary | | | 5 |
| Humanistic Care | Empathetic communication | | | 5 |

**Case10 Hematuria**

| **Content Structure** | **Inquiry Assessment Content** | | | **Points** |
| --- | --- | --- | --- | --- |
| Preparation and Introduction | Self-introduction | | | 1 |
|  | Explanation of duties | | | 1 |
|  | Obtaining patient cooperation | | | 1 |
| General data | 1.Patient's name | | | 0.5 |
|  | 2.Patient's gender | | | 0.5 |
|  | 3.Patient's age | | | 0.5 |
|  | 4.Patient's ethnicity | | | 0.5 |
|  | 5.Patient's native place | | | 0.5 |
|  | 6.Patient's birthplace | | | 0.5 |
|  | 7. Patient's current residence | | | 0.5 |
|  | 8.Patient's occupation | | | 0.5 |
|  | 9. Patient's marital status | | | 0.5 |
|  | 10. Patient's specific contact information | | | 0.5 |
| History of present illness | Chief complaints and characteristic | Hematuria | 1.Chief complaint symptom | 4 |
|  |  |  | 2.Onset time | 4 |
|  |  |  | 3.Onset speed | 2 |
|  |  |  | 4.Onset process of hematuria | 1 |
|  |  |  | Urine volume during hematuria | 1 |
|  |  |  | 5.Color and characteristics of urine during hematuria | 2 |
|  |  |  | 6.Urination frequency and number of times during hematuria | 2 |
|  |  |  | 7.Duration of hematuria | 2 |
|  |  |  | 8.Relationship between red-tinged urine and diet/medication | 1 |
|  |  |  | 9.Inducements of hematuria | 4 |
|  |  |  | 10.Factors aggravating or alleviating hematuria symptoms | 1 |
|  |  |  | 11.Development and evolution process of hematuria | 1 |
|  |  |  | 12.Previous episodes of hematuria | 1 |
|  |  |  | 13.Associated symptoms of hematuria | 2 |
|  |  |  | 14.Segmentation of urine color in hematuria | 2 |
|  | Accompanying Symptoms | Frequent Urination, Urgent Urination, and Dysuria (Positive) | 1.Confirmation of frequent urination, urgent urination, and dysuria symptoms | 1 |
|  |  |  | 2.Onset time of frequent urination, urgent urination, and dysuria | 0.5 |
|  |  |  | 3.Onset process of frequent urination, urgent urination, and dysuria | 0.5 |
|  |  |  | 4.Urine volume during frequent urination, urgent urination, and dysuria | 0.5 |
|  |  |  | 5.Color and characteristics of urine during frequent urination, urgent urination, and dysuria | 0.5 |
|  |  |  | 6.Duration of frequent urination, urgent urination, and dysuria | 0.5 |
|  |  |  | 7.Frequency of frequent urination, urgent urination, and dysuria symptoms | 0.5 |
|  |  |  | 8.Inducements of frequent urination, urgent urination, and dysuria symptoms | 0.5 |
|  |  |  | 9.Factors aggravating or alleviating frequent urination, urgent urination, and dysuria symptoms | 0.5 |
|  |  |  | 10.evelopment and evolution process of frequent urination, urgent urination, and dysuria | 0.5 |
|  |  |  | 11.Previous episodes of frequent urination, urgent urination, and dysuria | 0.5 |
|  |  |  | 12.Associated symptoms of frequent urination, urgent urination, and dysuria | 0.5 |
|  |  |  | 13.Location of dysuria | 0.5 |
|  |  | 1.Low Back Pain (Negative) | 1.Confirmation of low back pain symptoms | 1 |
|  |  | 2.Fever (Negative) | 1.Confirmation of fever symptoms | 1 |
|  |  | 3.Dizziness (Negative) | 1.Confirmation of dizziness symptoms | 1 |
|  | **Diagnostic and Therapeutic Process** | Emergency treatment | | 1 |
|  |  | Previous medical treatment unit | | 1 |
|  |  | Previous examination results | | 1 |
|  |  | Medication history | | 1 |
|  | Current General Condition | Appetite | | 1 |
|  |  | Defecation and urination | | 1 |
|  |  | Weight changes | | 1 |
|  |  | Mental state | | 1 |
|  |  | Sleep quality | | 1 |
|  |  | Physical strength | | 1 |
| History of past illness | 1.Chronic disease history | 1.Confirmation of other previous diseases | | 2 |
|  |  | 2.Long-term medication history | | 1 |
|  | 2.Infectious disease history | 1.History of infectious diseases | | 1 |
|  | 3.Surgical and traumatic history | 1.Traumatic history | | 1 |
|  |  | 2.Surgical history | | 1 |
|  | 4.Allergy history | 1.Allergy history | | 1 |
|  | 5.Vaccination history | 1.Vaccination history | | 1 |
|  | 6.Blood transfusion history | 1.Whether there has been a blood transfusion | | 1 |
| Personal History | 1.Social experiences | 1.Long-term residence area | | 0.5 |
|  |  | 2.Epidemic focus and endemic area | | 0.5 |
|  |  | 3.Epidemiological history | | 0.5 |
|  | 2.Habits and hobbies | 1.Alcohol consumption history | | 0.5 |
|  |  | 2.Smoking history | | 0.5 |
|  | 3.Occupational working conditions | 1.Occupational type | | 0.5 |
|  |  | 2.Occupation and working conditions | | 0.5 |
|  |  | 3.Work labor intensity | | 0.5 |
|  | 4.Sexual promiscuity history | 1.History of unclean sexual intercourse | | 0.5 |
|  |  | 2.History of sexually transmitted diseases | | 0.5 |
| Marital and Reproductive History | 1.Marital status | 1.Spouse's physical health status | | 0.5 |
|  |  | 2.Age at marriage | | 0.5 |
|  |  | 3.Whether married | | 0.5 |
|  | 2.Children's information | 1.Children's information | | 0.5 |
|  | 3.Menstrual History | 1.Age at menarche | | 0.5 |
|  |  | 2.Menstrual cycle | | 0.5 |
|  |  | 3.Duration of menstrual flow | | 0.5 |
|  |  | 4Time of last menstrual period | | 0.5 |
|  |  | 5.Volume of last menstrual period | | 0.5 |
|  |  | 6.Duration of last menstrual period | | 0.5 |
| Family History | 1.Family medical history | 1.Family genetic disease history | | 1 |
|  |  | 2.Similar family medical history | | 1 |
|  |  | 3.Parents' health conditions | | 1 |
| Inquiry Skills | Inquiry sequence | | | 5 |
|  | Communication skills | | | 5 |
|  | Complex professional vocabulary | | | 5 |
| Humanistic Care | Empathetic communication | | | 5 |

**Case 11 Emaciation**

| **Content Structure** | **Inquiry Assessment Content** | | | **Points** |
| --- | --- | --- | --- | --- |
| Preparation and Introduction | Self-introduction | | | 1 |
|  | Explanation of duties | | | 1 |
|  | Obtaining patient cooperation | | | 1 |
| General data | 1.Patient's name | | | 0.5 |
|  | 2.Patient's gender | | | 0.5 |
|  | 3.Patient's age | | | 0.5 |
|  | 4.Patient's ethnicity | | | 0.5 |
|  | 5.Patient's native place | | | 0.5 |
|  | 6.Patient's birthplace | | | 0.5 |
|  | 7. Patient's current residence | | | 0.5 |
|  | 8.Patient's occupation | | | 0.5 |
|  | 9. Patient's marital status | | | 0.5 |
|  | 10. Patient's specific contact information | | | 0.5 |
| History of present illness | Chief complaints and characteristic | Emaciation | 1.Chief complaint symptom | 4 |
|  |  |  | 2.Onset urgency | 2 |
|  |  |  | 3.Onset time of emaciation | 4 |
|  |  |  | 4.Degree of emaciation | 4 |
|  |  |  | 5.Relationship between emaciation and diet/exercise | 3 |
|  |  |  | 6.Relationship between emaciation and medication intake | 3 |
|  |  |  | 7.Development and evolution process of emaciation | 4 |
|  |  |  | 8.Previous episodes of emaciation | 2 |
|  |  |  | 9.Inducing factors for the onset of emaciation | 4 |
|  | Accompanying Symptoms | **1.Palpitations (Positive)** | 1.Confirmation of palpitation symptom | 0.5 |
|  |  |  | 2.Onset time of palpitations | 0.5 |
|  |  |  | 3.Intensity of palpitations | 0.5 |
|  |  |  | 4.Duration of palpitations | 0.5 |
|  |  |  | 5.Etiological factors of palpitations | 0.5 |
|  |  |  | 6.Aggravating or relieving factors of palpitations | 0.5 |
|  |  | **2.Hand and Foot Tremors (Positive)** | 1.Confirmation of hand and foot tremor symptom | 0.5 |
|  |  |  | 2.Onset and duration of hand and foot tremors | 0.5 |
|  |  |  | 3.Location of hand and foot tremor symptoms | 0.5 |
|  |  |  | 4.Nature of hand and foot tremor symptoms | 0.5 |
|  |  | **1.Fever (Negative)** | 1.Confirmation of fever symptom | 1 |
|  |  | **2.Nausea and Vomiting (Negative)** | 1.Confirmation of nausea and vomiting symptoms | 1 |
|  |  | **3.Diarrhea (Negative)** | 1.Confirmation of diarrhea symptom | 1 |
|  |  | **4.Polyphagia, Polydipsia, and Polyuria** | 1.Confirmation of polyphagia, polydipsia, and polyuria symptoms | 1 |
|  |  | **5.Night Sweats (Negative)** | 1.Confirmation of night sweat symptom | 1 |
|  | **Diagnostic and Therapeutic Process** | 1.Previous examination results | | 2 |
|  |  | 2.Medication usage | | 2 |
|  | Current General Condition | Appetite | | 1 |
|  |  | Defecation and urination | | 1 |
|  |  | Weight changes | | 1 |
|  |  | Mental state | | 1 |
|  |  | Sleep quality | | 1 |
|  |  | Physical strength | | 1 |
| History of past illness | 1.Chronic disease history | 1.Confirmation of other previous diseases | | 2 |
|  | 2.Infectious disease history | 1.History of exposure to infectious diseases | | 1 |
|  |  | 2.History of infectious diseases | | 1 |
|  | 3.Surgical and traumatic history | 1.Traumatic history | | 1 |
|  |  | 2.Surgical history | | 1 |
|  | 4.Allergy history | 1.Allergy history | | 1 |
|  | 5.Vaccination history | 1.Vaccination history | | 1 |
|  | 6.Blood transfusion history | 1.Whether there has been a blood transfusion | | 1 |
| Personal History | 1.Social experiences | 1.Long-term residence area | | 0.5 |
|  |  | 2.Epidemic focus and endemic area | | 0.5 |
|  | 2.Occupational working conditions | 1.Occupational type | | 0.5 |
|  |  | 2.Working conditions and environment | | 0.5 |
|  | 3.Habits and hobbies | 1.Alcohol consumption history | | 1 |
|  |  | 2.Smoking history | | 1 |
|  | 4.Sexual promiscuity history | 1.History of unclean sexual intercourse | | 1 |
| Marital and Reproductive History | 1.Marital status | 1.Whether married | | 5 |
| Family History | 1.Family medical history | 1.Family genetic disease history | | 1 |
|  |  | 2.Similar family medical history | | 1 |
|  |  | 3.Parents' health conditions | | 1 |
| Inquiry Skills | Inquiry sequence | | | 5 |
|  | Communication skills | | | 5 |
|  | Complex professional vocabulary | | | 5 |
| Humanistic Care | Empathetic communication | | | 5 |

**Scoring Rubrics for Baseline Test**

**Fever**

| **Content Structure** | **Inquiry Assessment Content** | | | **Points** |
| --- | --- | --- | --- | --- |
| Preparation and Introduction | Self-introduction | | | 1 |
|  | Explanation of duties | | | 1 |
|  | Obtaining patient cooperation | | | 1 |
| General data | 1.Patient's name | | | 0.5 |
|  | 2.Patient's gender | | | 0.5 |
|  | 3.Patient's age | | | 0.5 |
|  | 4.Patient's ethnicity | | | 0.5 |
|  | 5.Patient's native place | | | 0.5 |
|  | 6.Patient's birthplace | | | 0.5 |
|  | 7. Patient's current residence | | | 0.5 |
|  | 8.Patient's occupation | | | 0.5 |
|  | 9. Patient's marital status | | | 0.5 |
|  | 10. Patient's specific contact information | | | 0.5 |
| History of present illness | Chief complaints and characteristic | Fever | 1.Chief complaint symptom | 4 |
|  |  |  | 2.Onset time | 4 |
|  |  |  | 3.Onset speed | 2 |
|  |  |  | 4.Etiological and inducible factors of fever | 4 |
|  |  |  | 5.Degree of fever | 2 |
|  |  |  | 6.Progression of the fever course | 2 |
|  |  |  | 7.Duration of fever | 2 |
|  |  |  | 8.Factors aggravating or alleviating fever | 3 |
|  |  |  | 9.Associated symptoms of fever | 3 |
|  |  |  | 10.Fever reduction situation | 2 |
|  |  |  | 11.Chills during fever | 2 |
|  | Accompanying Symptoms | 1.Cough and Sputum Expectoration (Positive) | 1.Confirmation of cough symptoms | 1 |
|  |  |  | 2.Onset time of cough | 1 |
|  |  |  | 3.Progression of the cough course | 1 |
|  |  |  | 4.Nature of cough | 1 |
|  |  |  | 5.Characteristics and volume of sputum | 1 |
|  |  |  | 6.Time pattern of cough | 1 |
|  |  |  | 7.Severity of cough onset | 1 |
|  |  | 1.Shortness of Breath (Negative) | 1.Confirmation of shortness of breath symptoms | 1 |
|  |  | 2.Hemoptysis (Negative) | 1.Confirmation of hemoptysis symptoms | 0.5 |
|  |  | 3.Palpitations (Negative) | 1.Confirmation of palpitations symptoms | 0.5 |
|  |  | 4.Nausea and Vomiting (Negative) | 1.Confirmation of nausea and vomiting symptoms | 1 |
|  | Diagnostic and Therapeutic Process | Previous medical treatment unit | | 1 |
|  |  | Previous examination results | | 1 |
|  |  | Previous medication names | | 0.5 |
|  |  | Previous medication time | | 0.5 |
|  |  | Previous medication dosage | | 0.5 |
|  |  | Efficacy of previous medications | | 0.5 |
|  | Current General Condition | Appetite | | 1 |
|  |  | Defecation and urination | | 1 |
|  |  | Weight changes | | 1 |
|  |  | Mental state | | 1 |
|  |  | Sleep quality | | 1 |
|  |  | Physical strength | | 1 |
| History of past illness | 1.Chronic disease history | 1.Confirmation of other previous diseases | | 2 |
|  |  | 2.Long-term medication history | | 1 |
|  | 2.Infectious disease history | 1.History of infectious diseases | | 1 |
|  | 3.Surgical and traumatic history | 1.Traumatic history | | 1 |
|  |  | 2.Surgical history | | 1 |
|  | 4.Allergy history | 1.Allergy history | | 1 |
|  | 5.Vaccination history | 1.Vaccination history | | 1 |
|  | 6.Blood transfusion history | 1.Whether there has been a blood transfusion | | 1 |
| Personal History | 1.Social experiences | 1.Long-term residence area | | 0.5 |
|  |  | 2.Epidemic focus and endemic area | | 0.5 |
|  | 2.Occupational working conditions | 1.Occupational working conditions | | 1 |
|  | 3.Habits and hobbies | 1.Alcohol consumption history | | 1 |
|  |  | 2.Smoking history | | 1 |
|  | 4.Sexual promiscuity history | 1.History of unclean sexual intercourse | | 1 |
| Marital and Reproductive History | 1.Marital status | 1.Whether married | | 5 |
| Family History | 1.Family medical history | 1.Family genetic disease history | | 1 |
|  |  | 2.Similar family medical history | | 1 |
|  |  | 3.Parents' health conditions | | 1 |
| Inquiry Skills | Inquiry sequence | | | 5 |
|  | Communication skills | | | 5 |
|  | Complex professional vocabulary | | | 5 |
| Humanistic Care | Empathetic communication | | | 5 |

**Chest Pain**

| **Content Structure** | **Inquiry Assessment Content** | | | **Points** |
| --- | --- | --- | --- | --- |
| Preparation and Introduction | Self-introduction | | | 1 |
|  | Explanation of duties | | | 1 |
|  | Obtaining patient cooperation | | | 1 |
| General data | 1.Patient's name | | | 0.5 |
|  | 2.Patient's gender | | | 0.5 |
|  | 3.Patient's age | | | 0.5 |
|  | 4.Patient's ethnicity | | | 0.5 |
|  | 5.Patient's native place | | | 0.5 |
|  | 6.Patient's birthplace | | | 0.5 |
|  | 7. Patient's current residence | | | 0.5 |
|  | 8.Patient's occupation | | | 0.5 |
|  | 9. Patient's marital status | | | 0.5 |
|  | 10. Patient's specific contact information | | | 0.5 |
| History of present illness | Chief complaints and characteristic | Chest Pain | 1.Chief complaint symptom | 4 |
|  |  |  | 2.Onset time | 4 |
|  |  |  | 3.Onset speed | 2 |
|  |  |  | 4.Etiological and inducible factors of chest pain | 4 |
|  |  |  | 5.Location of chest pain | 2 |
|  |  |  | 6.Scope of chest pain symptoms | 2 |
|  |  |  | 7.Severity of chest pain | 2 |
|  |  |  | 8.Duration of chest pain | 2 |
|  |  |  | 9.Nature of chest pain | 2 |
|  |  |  | 10.Factors aggravating or alleviating chest pain | 2 |
|  |  |  | 11.Previous episodes of chest pain | 2 |
|  |  |  | 12.Associated symptoms of chest pain | 2 |
|  | Accompanying Symptoms | 1.Nausea and Vomiting (Positive) | 1.Confirmation of nausea and vomiting symptoms | 2 |
|  |  | 1.Headache (Negative) | 1.Confirmation of headache symptoms | 1 |
|  |  | 2.Dizziness (Negative) | 1.Confirmation of dizziness symptoms | 1 |
|  |  | 3.Dyspnea (Negative) | 1.Confirmation of dyspnea symptoms | 2 |
|  |  | 4.Hemoptysis (Negative) | 1.Confirmation of hemoptysis symptoms | 1 |
|  |  | 5.Acid Regurgitation (Negative) | 1.Confirmation of acid regurgitation symptoms | 1 |
|  |  | 6.Cough and Sputum Expectoration (Negative) | 1.Confirmation of cough and sputum expectoration symptoms | 1 |
|  |  | 7.Fever (Negative) | 1.Confirmation of fever symptoms | 1 |
|  | **Diagnostic and Therapeutic Process** | Previous medical treatment unit | | 0.5 |
|  |  | Previous examination results | | 0.5 |
|  |  | Previous treatment measures | | 0.5 |
|  |  | Previous medication names | | 0.5 |
|  |  | Previous medication time | | 0.5 |
|  |  | Previous medication dosage | | 0.5 |
|  |  | Efficacy of previous medications | | 0.5 |
|  |  | Previous diagnostic results | | 0.5 |
|  | Current General Condition | Appetite | | 1 |
|  |  | Defecation and urination | | 1 |
|  |  | Weight changes | | 1 |
|  |  | Mental state | | 1 |
|  |  | Sleep quality | | 1 |
|  |  | Physical strength | | 1 |
| History of past illness | 1.Chronic disease history | 1.Confirmation of other previous diseases | | 1 |
|  |  | 2.Previous treatment history of chronic diseases | | 0.5 |
|  |  | 3.Previous control status of chronic diseases | | 0.5 |
|  |  | 4.Long-term medication history | | 1 |
|  | 2.Infectious disease history | 1.History of infectious diseases | | 1 |
|  | 3.Surgical and traumatic history | 1.Traumatic history | | 1 |
|  |  | 2.Surgical history | | 1 |
|  | 4.Allergy history | 1.Allergy history | | 1 |
|  | 5.Vaccination history | 1.Vaccination history | | 1 |
|  | 6.Blood transfusion history | 1.Whether there has been a blood transfusion | | 1 |
| Personal History | 1.Social experiences | 1.Long-term residence area | | 0.5 |
|  |  | 2.Epidemic focus and endemic area | | 0.5 |
|  | 2.Occupation and working conditions | 1.Occupation and working conditions | | 1 |
|  | 3.Habits and hobbies | 1.Alcohol consumption history | | 0.5 |
|  |  | 2.Smoking history | | 0.5 |
|  |  | 3.Smoking start time | | 0.5 |
|  |  | 4.Smoking amount | | 0.5 |
|  | 4.Sexual promiscuity history | 1.History of unclean sexual intercourse | | 1 |
| Marital and Reproductive History | 1.Marital status | 1.Whether married | | 5 |
| Family History | 1.Family medical history | 1.Family genetic disease history | | 1 |
|  |  | 2.Similar family medical history | | 1 |
|  |  | 3.Parents' health conditions | | 1 |
| Inquiry Skills | Inquiry sequence | | | 5 |
|  | Communication skills | | | 5 |
|  | Complex professional vocabulary | | | 5 |
| Humanistic Care | Empathetic communication | | | 5 |
