# Supplementary material for: Effect of Large Language Model–Powered Virtual Standardized Patients on History-Taking Among Undergraduate Medical Students: Propensity-Matched Cohort Study
Source: JMIR Med Educ. 2026 Sep 9;12:e92486. doi: 10.2196/92486 (PMC13601875; doi:10.2196/92486)
Supplement: Supplementary file 3 [file mededu_v12i1e92486_app3.docx]

Yes

**Final**

**Control Group**

**Group B (n=48)**

**Final**

**Experimental Group Group (n=120)**

Re-assigned to

Control Group

(n=9)

Provisional

Control Group (n=39)

Provisional

Experimental Group (n=129)

Voluntarily registered for LLM-VSPpractice

**Initial Cohort**

**Third-year medical students**

**(n=276)**

Excluded (n=108)

- failed to complete the baseline test

(n=107)

- failed to complete the final test

(n=1)

Students included in data analysis (n=168)

minimum effective practice threshold:

- singal practice session ≥4 min
- AI score ≥ 25 points

Eligibility Assessment

Yes

No

No
