# Supplementary material for: Effect of Large Language Model–Powered Virtual Standardized Patients on History-Taking Among Undergraduate Medical Students: Propensity-Matched Cohort Study
Source: JMIR Med Educ. 2026 Sep 9;12:e92486. doi: 10.2196/92486 (PMC13601875; doi:10.2196/92486)
Supplement: Supplementary file 4 [file mededu_v12i1e92486_app4.docx]

**Appendix 4**

**Scoring Rubrics for Final Medical History-taking Test**

**Case1 Pneumonia**

| **Primary Category** | **Scoring Details** | **Points** |
| --- | --- | --- |
| Content Assessment | Physician self-introduction | 2 |
|  | Physician asks patient's name and age | 2 |
|  | Physician asks patient's general information | 2 |
|  | Time of onset: 7 days | 4 |
|  | Precipitating factor: Catching a cold | 4 |
|  | Symptom characteristics: Fever, cough, sputum production | 4 |
|  | Fever pattern: Persistent | 4 |
|  | Cough pattern: Paroxysmal, irritative | 4 |
|  | Sputum characteristics: Yellow viscous sputum, scant amount initially, gradually increasing | 4 |
|  | Negative symptoms: No nausea/vomiting, no abdominal distension/jaundice, no palpitations/shortness of breath, no urinary frequency/urgency/pain | 4 |
|  | Mental status, sleep, bowel and urinary habits, weight changes since onset | 2 |
|  | Past medical history | 4 |
|  | Personal history | 4 |
|  | Marital and reproductive history | 2 |
|  | Family history | 2 |
|  | Summarize patient's chief complaint: Fever, cough, sputum production for 7 days | 4 |
|  | Discuss possible diagnosis with patient: Pneumonia | 4 |
|  | Discuss next steps for examination and preliminary management: Urine/stool tests, liver/kidney function/electrolyte tests, sputum culture, chest X-ray. Anti-inflammatory, symptomatic treatment | 4 |
| Technique Assessment | Well-organized, clear purpose of questions, focused emphasis | 4 |
|  | Sequential questioning, clear questions: Starting with general questions (e.g., "What discomfort do you have?") or direct questions (e.g., "How long have you been unwell?"), no leading or challenging questions | 4 |
|  | Use of summarization skills, verifying information provided by patient | 4 |
|  | Appropriate use of transitional phrases | 4 |
|  | Use of plain language, avoiding medical jargon; if terminology is used, explain to patient | 4 |
|  | Examiner actively encourages patient questions to obtain more information and clarify existing information | 4 |
|  | Examiner provides satisfactory responses, understands patient's concerns, and provides sufficient information | 4 |
|  | Examiner listens attentively, uses encouraging phrases appropriately (e.g., "Please continue, I understand"), allows patient to describe history without interruption, uses pauses appropriately | 4 |
|  | Correct use of body language, e.g., appropriate eye contact | 4 |
|  | Neat appearance, respects patient, gains trust, shows empathy, makes patient feel comfortable, establishes good doctor-patient relationship | 4 |

**Case2 Coronary heart disease**

| **Primary Classification** | **Scoring Details** | **Ponits** |
| --- | --- | --- |
| Content Assessment | Physician self-introduction | 2 |
|  | Physician asks patient's name and age | 2 |
|  | Physician asks patient's general information | 2 |
|  | Time of onset: Three hours | 4 |
|  | Precipitating factor: None | 4 |
|  | Location of chest pain: Precordial area | 4 |
|  | Duration of chest pain: 15 minutes | 4 |
|  | Character of chest pain: Dull pain, crushing sensation, gradually worsening | 4 |
|  | Radiation: Throat tightness and back distending pain | 4 |
|  | Associated symptoms and negative symptoms: Profuse sweating | 4 |
|  | Mental status, diet, sleep, bowel and urinary habits, weight changes since onset | 2 |
|  | Past medical history: Previous hypertension for 10 years, highest BP 160/100mmHg | 4 |
|  | Personal history: Smoking for over 20 years, 2 packs/day, no alcohol consumption | 4 |
|  | Marital and reproductive history: Married at appropriate age, has one son and one daughter, spouse and children are healthy | 2 |
|  | Family history: Parents alive, no family history of genetic diseases | 2 |
|  | Summarize patient's chief complaint: Sudden chest pain for 3 hours | 4 |
|  | Discuss possible diagnosis with patient: Coronary heart disease, Acute coronary syndrome | 4 |
|  | Discuss next steps for examination and preliminary management: Routine blood/urine/stool tests, liver/kidney function/electrolyte tests, myocardial biomarker tests, cardiac ultrasound, coronary angiography. Antiplatelet therapy, anti-myocardial ischemia therapy, lipid-lowering therapy, myocardial protection therapy. | 4 |
| Technique Assessment | Well-organized, clear purpose of questions, focused emphasis | 4 |
|  | Sequential questioning, clear questions: Starting with general questions (e.g., "What discomfort do you have?") or direct questions (e.g., "How long have you had the pain?"), no leading or challenging questions | 4 |
|  | Use of summarization skills, verifying information provided by patient | 4 |
|  | Appropriate use of transitional phrases | 4 |
|  | Use of plain language, avoiding medical jargon; if terminology is used, explain to patient | 4 |
|  | Examiner actively encourages patient questions to obtain more information and clarify existing information | 4 |
|  | Examiner provides satisfactory responses, understands patient's concerns, and provides sufficient information | 4 |
|  | Examiner listens attentively, uses encouraging phrases appropriately (e.g., "Please continue, I understand"), allows patient to describe history without interruption, uses pauses appropriately | 4 |
|  | Correct use of body language, e.g., appropriate eye contact | 4 |
|  | Neat appearance, respects patient, gains trust, shows empathy, makes patient feel comfortable, establishes good doctor-patient relationship | 4 |

**Case 3 Hyperthyroidism**

| **Primary Classification** | **Scoring Details** | **Points** |
| --- | --- | --- |
| Content Assessment | Physician self-introduction | 2 |
|  | Physician asks patient's name and age | 2 |
|  | Physician asks patient's general information | 2 |
|  | Time of onset: One year | 4 |
|  | Precipitating factor: None | 4 |
|  | Symptom characteristics: Palpitations, heat intolerance, excessive sweating, significantly increased appetite, weight loss of about 5 kg | 4 |
|  | Cardiac manifestations: Palpitations, rapid heartbeat, especially noticeable at night | 4 |
|  | Endocrine and metabolic symptoms: Heat intolerance, excessive sweating, weight loss | 2 |
|  | Neuropsychiatric symptoms: Nervousness, anxiety, talkativeness, hyperactivity, insomnia, irritability | 2 |
|  | Musculoskeletal system: Fatigue, weakness, numbness | 2 |
|  | Digestive system symptoms: Diarrhea or loose stools | 2 |
|  | Disease progression: Gradually worsened over the past three weeks | 2 |
|  | History of diagnosis and treatment | 2 |
|  | Mental status, diet, sleep, bowel and urinary habits since onset | 2 |
|  | Past medical history | 4 |
|  | Personal history | 4 |
|  | Menstrual, marital and reproductive history | 2 |
|  | Family history | 2 |
|  | Summarize patient's chief complaint: Heat intolerance, excessive sweating, palpitations, increased appetite for one year, worsened for three weeks | 4 |
|  | Discuss possible diagnosis with patient: Hyperthyroidism | 4 |
|  | Discuss next steps for examination and preliminary management: Routine blood/urine/stool tests, liver/kidney function/electrolyte tests, thyroid function tests, thyroid ultrasound. Antithyroid medication treatment. | 4 |
| Technique Assessment | Well-organized, clear purpose of questions, focused emphasis | 4 |
|  | Sequential questioning, clear questions: Starting with general questions (e.g., "What discomfort do you have?") or direct questions (e.g., "How long have you been unwell?"), no leading or challenging questions | 4 |
|  | Use of summarization skills, verifying information provided by patient | 4 |
|  | Appropriate use of transitional phrases | 4 |
|  | Use of plain language, avoiding medical jargon; if terminology is used, explain to patient | 4 |
|  | Examiner actively encourages patient questions to obtain more information and clarify existing information | 4 |
|  | Examiner provides satisfactory responses, understands patient's concerns, and provides sufficient information | 4 |
|  | Examiner listens attentively, uses encouraging phrases appropriately (e.g., "Please continue, I understand"), allows patient to describe history without interruption, uses pauses appropriately | 4 |
|  | Correct use of body language, e.g., appropriate eye contact | 4 |
|  | Neat appearance, respects patient, gains trust, shows empathy, makes patient feel comfortable, establishes good doctor-patient relationship | 4 |

**Case 4 Urinary tract infection**

| **Primary Category** | **Scoring Details** | **Points** |
| --- | --- | --- |
| Content Assessment | Physician self-introduction | 2 |
|  | Physician asks patient's name and age | 2 |
|  | Physician asks patient's general information | 2 |
|  | Time of onset: One week | 4 |
|  | Precipitating factor: None | 4 |
|  | Symptom characteristics: Dysuria, urinary frequency, urinary urgency | 4 |
|  | Location of dysuria: Urethral orifice | 4 |
|  | Urinary frequency: Time interval between urination | 4 |
|  | Urinary urgency: Incomplete emptying sensation, urine volume each time | 4 |
|  | Negative symptoms: Hematuria, pyuria | 4 |
|  | Mental status, diet, sleep, bowel habits since onset | 2 |
|  | Past medical history | 4 |
|  | Personal history | 4 |
|  | Menstrual, marital and reproductive history | 2 |
|  | Family history | 2 |
|  | Summarize patient's chief complaint: Dysuria, urinary frequency, urinary urgency for one week | 4 |
|  | Discuss possible diagnosis with patient: Urinary tract infection | 4 |
|  | Discuss next steps for examination and preliminary management: Complete blood count, urinalysis, stool routine, liver/kidney function/electrolyte tests, renal ultrasound, urine culture. Antibiotic treatment. | 4 |
| Technique Assessment | Well-organized, clear purpose of questions, focused emphasis | 4 |
|  | Sequential questioning, clear questions: Starting with general questions (e.g., "What discomfort do you have?") or direct questions (e.g., "How long have you been unwell?"), no leading or challenging questions | 4 |
|  | Use of summarization skills, verifying information provided by patient | 4 |
|  | Appropriate use of transitional phrases | 4 |
|  | Use of plain language, avoiding medical jargon; if terminology is used, explain to patient | 4 |
|  | Examiner actively encourages patient questions to obtain more information and clarify existing information | 4 |
|  | Examiner provides satisfactory responses, understands patient's concerns, and provides sufficient information | 4 |
|  | Examiner listens attentively, uses encouraging phrases appropriately (e.g., "Please continue, I understand"), allows patient to describe history without interruption, uses pauses appropriately | 4 |
|  | Correct use of body language, e.g., appropriate eye contact | 4 |
|  | Neat appearance, respects patient, gains trust, shows empathy, makes patient feel comfortable, establishes good doctor-patient relationship | 4 |
