# Supplementary material for: Effect of Large Language Model–Powered Virtual Standardized Patients on History-Taking Among Undergraduate Medical Students: Propensity-Matched Cohort Study
Source: JMIR Med Educ. 2026 Sep 9;12:e92486. doi: 10.2196/92486 (PMC13601875; doi:10.2196/92486)
Supplement: Supplementary file 5 [file mededu_v12i1e92486_app5.docx]

**Multimedia Appendix 5**

**Supplementary Statistical Analyses**

Table S1. ICC for the final MHT assessment scores across different stations

| Exam station | ICC | 95% CI |
| --- | --- | --- |
| Pneumonia | 0.77 | (0.39-0.90) |
| Coronary Heart Disease | 0.80 | (0.60-0.90) |
| Hyperthyroidism | 0.65 | (0.12-0.85) |
| Urinary Tract Infection | 0.78 | (0.60-0.88) |

Table S2. Comparison of Core Metrics between Primary Analysis (PP) and Sensitivity Analysis (ITT)

| Analytic Strategy | Matched Pairs (n) | Mean Difference in Total Score (95% CI) | Adjusted Regression Coefficient *B*  (95% CI) | Cohen *d* (95% CI) | Rosenbaum Bounds (Γ) |
| --- | --- | --- | --- | --- | --- |
| Primary Analysis (Per-Protocol set) | 40 | 3.98  (0.55-7.40) | 3.924  (1.904-5.944) | 0.52  (0.06-0.97) | 1.21 |
| Sensitivity Analysis (Intention-to-Treat set) | 33 | 5.27  (1.52-9.02) | 5.190  (2.505-7.875) | 0.70  (0.20-1.27) | 1.42 |

Table S3. Effective practice variables of LLM-VSPs-based training and their correlations with final MHT test scores in Group A (n=120)

| **Practice behavior data** | Mean (SD) | **Unadjusted**  **Correlation coefficient (*r*)**  **(95% CI)** | *P* | **Adjusted**  **Correlation coefficient (*r*)**  **(95% CI)** | *P* |
| --- | --- | --- | --- | --- | --- |
| Total number of effective practice sessions for LLM-VSPs-based MHT | 6.25 (SD 3.69) | 0.156  (-0.024 to 0.326) | .09 | 0.069  (-0.112 to 0.246) | .46 |
| Total number of effective practice cases for LLM-VSPs-based MHT | 5.21 (SD 3.07) | 0.153  (-0.027 to 0.323) | .10 | 0.071  (-0.110 to 0.247) | .44 |
| Average duration per practice session for LLM-VSPs-based MHT (min/each) | 17.08 (SD 4.51) | 0.113  (-0.068 to 0.286) | .22 | 0.021  (-0.159 to 0.200) | .82 |
| Total duration of LLM-VSPs-based MHT practice sessions (min) | 108.63 (SD 72.20) | 0.183  (0.004 to 0.351) | .046 | 0.075  (-0.106 to 0.251) | .42 |
| Highest AI-generated assessment score for LLM-VSPs-based MHT | 77.30 (SD 10.33) | 0.171  (-0.009 to 0.340) | .06 | -0.009  (-0.189 to 0.171) | .93 |
| Average AI-generated assessment score for LLM-VSPs-based MHT | 68.02 (SD 9.55) | 0.233  (0.056 to 0.396) | .01 | 0.097  (-0.084 to 0.273) | .29 |

Table S4. General Linear Model Analysis of Intervention Effects and Baseline Heterogeneity (Matched Sample, n=80)

| Variables / Effects | B (SE) | t | F (df1, df2) | 95% CI | P | Partial η² |
| --- | --- | --- | --- | --- | --- | --- |
| Panel A: Test of heterogeneity & covariate effects | | | | | | |
| Group × Baseline Score | -0.052 (0.106) | -0.489 | - | (-0.262 to 0.159) | .63 | 0.003 |
| Baseline Score | 0.044 (0.083) | 0.535 | - | (-0.120 to 0.209) | .59 | 0.004 |
| Final Test Case | - | - | 40.40 (3, 70) | - | <.001 | 0.634 |
| Gender | - | - | 8.81 (1, 70) | - | .004 | 0.112 |
| Academic Major | - | - | 3.10 (2, 70) | - | .051 | 0.081 |
| Panel B: Test of main intervention effect | | | | | | |
| Group A | 85.44 (0.95) | - | - | (83.54 to 87.34) | - | - |
| Group B | 81.52 (1.02) | - | - | (79.49 to 83.55) | - | - |
| Mean Difference (A - B) | 3.92 (1.02) | - | 14.81 (1, 70) | (1.89 to 5.95) | <.001 | 0.175 |

**Note: The model adjusted for gender, academic major, final test case, and baseline score (continuous).**

Table S5. General Linear Model Analysis of Intervention Effects and Baseline Heterogeneity (Overall Unmatched Sample, N=168)

| Variables / Effects | *B* (SE) | *t* | *F* (df1, df2) | 95% CI | *P* | Partial η² |
| --- | --- | --- | --- | --- | --- | --- |
| **Panel A: Test of heterogeneity & covariate effects** | | | | | | |
| Group × Baseline Score | -0.109 (0.084) | -1.292 | - | (-0.275 to 0.058) | .20 | 0.010 |
| Baseline Score | 0.128 (0.048) | 2.649 | - | (0.033 to 0.223) | .009 | 0.043 |
| Final Test Case | - | - | 40.92 (3, 158) | - | <.001 | 0.437 |
| Gender | - | - | 8.96 (1, 158) | - | .003 | 0.054 |
| Academic Major | - | - | 10.12 (2, 158) | - | <.001 | 0.114 |
| **Panel B: Test of main intervention effect** | | | | | | |
| Group A (Intervention) | 84.36 (0.59) | - | - | (83.19 to 85.52) | - | - |
| Group B (Control) | 80.75 (0.96) | - | - | (78.85 to 82.64) | - | - |
| Mean Difference (A - B) | **3.61 (0.99)** | - | **13.26 (1, 158)** | **(1.65** to **5.57)** | **<.001** | **0.077** |

**Note: The model adjusted for gender, academic major, final test case, and baseline score (continuous).**
